# DJ-1 Proteoforms in Breast Cancer Cells: The Escape of Metabolic Epigenetic Misregulation

**DOI:** 10.3390/cells9091968

**Published:** 2020-08-26

**Authors:** Domenica Scumaci, Erika Olivo, Claudia Vincenza Fiumara, Marina La Chimia, Maria Teresa De Angelis, Sabrina Mauro, Giosuè Costa, Francesca Alessandra Ambrosio, Stefano Alcaro, Valter Agosti, Francesco Saverio Costanzo, Giovanni Cuda

**Affiliations:** 1 Laboratory of Proteomics, Research Center on Advanced Biochemistry and Molecular Biology, Department of Experimental and Clinical Medicine, Magna Græcia Universityof Catanzaro, S Venuta University Campus, 88100 Catanzaro, Italy; erika.olivo26@gmail.com (E.O.); fiumara@unicz.it (C.V.F.); marina.lachi@gmail.com (M.L.C.); sabry93cz@gmail.com (S.M.); cuda@unicz.it (G.C.); 2 Stem Cell Laboratory, Research Center of Advanced Biochemistry and Molecular Biology, Department of Experimental and Clinical Medicine, University Magna Graeciaof Catanzaro, S. Venuta University Campus, 88100 Catanzaro, Italy; mariateresadeangelis211285@gmail.com; 3 Department of Health Sciences, University Magna Græcia of Catanzaro, Campus S. Venuta, 88100 Catanzaro, Italy; gcosta@unicz.it (G.C.); ambrosio@unicz.it (F.A.A.); alcaro@unicz.it (S.A.); 4 Net4Science Academic Spin-Off, University Magna Græcia of Catanzaro, Campus S. Venuta, Viale Europa, 88100 Catanzaro, Italy; 5 Laboratory of Molecular Oncology, Department of Experimental and Clinical Medicine, CIS for Genomics and Molecular Pathology, Magna Græcia University of Catanzaro, 88100 Catanzaro, Italy; agosti@unicz.it (V.A.); fsc@unicz.it (F.S.C.)

**Keywords:** metabolic rewiring, DJ-1, AGEs, Akt, breast cancer, histones, 2D TAU gel, glycation

## Abstract

Enhanced glycolysis is a hallmark of breast cancer. In cancer cells, the high glycolytic flux induces carbonyl stress, a damaging condition in which the increase of reactive carbonyl species makes DNA, proteins, and lipids more susceptible to glycation. Together with glucose, methylglyoxal (MGO), a byproduct of glycolysis, is considered the main glycating agent. MGO is highly diffusible, enters the nucleus, and can react with easily accessible lysine- and arginine-rich tails of histones. Glycation adducts on histones undergo oxidization and further rearrange to form stable species known as advanced glycation end-products (AGEs). This modification alters nucleosomes stability and chromatin architecture deconstructing the histone code. Formation of AGEs has been associated with cancer, diabetes, and several age-related diseases. Recently, DJ-1, a cancer-associated protein that protects cells from oxidative stress, has been described as a deglycase enzyme. Although its role in cell survival results still controversial, in several human tumors, its expression, localization, oxidation, and phosphorylation were found altered. This work aimed to explore the molecular mechanism that triggers the peculiar cellular compartmentalization and the specific post-translational modifications (PTM) that, occurring in breast cancer cells, influences the DJ-1 dual role. Using a proteomic approach, we identified on DJ-1 a novel threonine phosphorylation (T125) that was found, by the in-silico tool scansite 4, as part of a putative Akt consensus. Notably, this threonine is in addition to histidine 126, a key residue involved in the formation of catalytic triade (glu18-Cys106-His126) inside the glioxalase active site of DJ. Interestingly, we found that pharmacological modulation of Akt pathway induces a functional tuning of DJ-1 proteoforms, as well as their shuttle from cytosol to nucleus, pointing out that pathway as critical in the development of DJ-1 pro-tumorigenic abilities. Deglycase activity of DJ-1 on histones proteins, investigated by coupling 2D tau gel with LC-MS/MS and 2D-TAU (Triton-Acid-Urea)-Western blot, was found correlated with its phosphorylation status that, in turn, depends from Akt activation. In normal conditions, DJ-1 acts as a redox-sensitive chaperone and as an oxidative stress sensor. In cancer cells, glycolytic rewiring, inducing increased reactive oxygen species (ROS) levels, enhances AGEs products. Alongside, the moderate increase of ROS enhances Akt signaling that induces DJ-1-phosphorylation. When phosphorylated DJ-1 increases its glyoxalase activity, the level of AGEs on histones decreases. Therefore, phospho-DJ-1 prevents glycation-induced histones misregulation and its Akt-related hyperactivity represents a way to preserve the epigenome landscape sustaining proliferation of cancer cells. Together, these results shed light on an interesting mechanism that cancer cells might execute to escape the metabolic induced epigenetic misregulation that otherwise could impair their malignant proliferative potential.

## 1. Introduction

Metabolic rewiring is the main hallmark of several types of cancer. Cancer cells rewire their metabolic program to meet the energetic requirements sustaining proliferation, survival, and invasion. The comprehension of the pathway triggering metabolic changes, that sustain the high energetic and anabolic requirements of the malignant phenotype, might greatly improve the understanding of tumor biology and the development of targeted therapies.

In breast cancer cells, the Warburg effect is a common feature, shared by all breast cancer subtypes [1]. The switch toward aerobic glycolysis accounts for the production of biohazard products as Glyoxal and Methylglyoxal (MGO) able to react with peculiar amino acids, including arginines and lysines. The reaction goes through the formation of Maillard adducts that are finally transformed into advanced glycation end products (AGEs). Histone proteins are the main target of this reaction because of the reactive amino acids forming the histone tails and due to the high diffusibility of MGO.

Histones are the main component of nucleosome, the molecular structure in which DNA is packaged in the nucleus. DNA is wrapped on histones core and the structure is stabilized by histone linker H1. Histones tails are rich in lysine and arginine, they protrude away from the nucleosome core and might undergo several post-translational modifications (PTMs), e.g., phosphorylation acetylation and methylation. PTMs are key factors in tuning the accessibility of chromatin to transcription factors.

The plethora of histones PTMs represents the “Histone Code”, the “handbook” that cells use to correctly replicate a propagate. Alterations on Histone Code was related with several types of tumors, including breast cancer [2].

The formation of histones AGEs represents, for living cells, a catastrophic event as they induce the destruction of histone code triggering senescence. It was reported that cancer cells overexpress DJ-1 a deglycases able to remove AGEs from histones proteins and preserve cell survival [3,4].

DJ-1, a 20 kDa protein with a well-conserved sequence [5], is a multifunctional protein. It acts as redox-regulate chaperone, cysteine protease, regulator of RNA-binding protein, and transcriptional coactivator [5,6,7,8] acting on several biological processes, including antioxidant stress function and mitochondrial regulation [9,10,11]. DJ-1 is overexpressed in several tumor types, including breast cancer [4]. In cancer cells, DJ-1 is required for maintenance of the transformed phenotype, being implicated in growth, survival, and chemoresistance [12].

In this work, we applied a previously developed proteomic strategy [13] to map AGEs formation on histone in breast cancer cells and to detect a novel phosphorylation on DJ-1 protein accountable for the modulation of its glyoxalase activity. “Proteomics approaches are unrivaled by other technologies, as enables not only the identification and quantification of proteins, but also the determination of their modifications, localization, interactions, and ultimately, their function” [14].

We introduce a new order of complexity for DJ-1 protein function, providing evidence that the glyoxalase activity, determinant to counteract aging triggered by AGE formation on histone proteins, may be modulated by mitogenic pathway. We show that the novel phosphorylation on DJ-1 may neutralize the impairment of AGEs formations on histones, leading to the inactivation of senescence programs. Exploiting the potential of this PTM in tumor biology may, therefore, represents a useful tool to direct cancer cells survival.

## 2. Materials and Methods

### 2.1. Cells Culture

MCF7 (HTB-22 from ATCC, Manassas, VA, USA), a model of human sporadic breast cancer cell line, luminal A, were grown in Dulbecco’s modified Eagle’s medium (DMEM) (Sigma Aldrich, Saint Louis, MO, USA, implemented with 100 mg/mL streptomycin and 100 U/mL penicillin (Sigma Aldrich) and 10% (*w*/*v*) fetal bovine serum (FBS) (Sigma Aldrich). MCF10 are a model of immortalized mammary epithelial cells (CRL-10317 from ATCC, Manassas, VA, USA). Cells were grown in MEGM, Mammary Epithelial Cell Growth Medium (Lonza, Walkersville, MD, USA), implemented with 20 ng/mL epidermal growth factor (Lonza),10 μg/mL insulin (Lonza), 0.5 μg/mL hydrocortisone (Lonza), and 100 ng/mL cholera toxin (Sigma Aldrich). The HCC1937 (CRL-2336 from ATCC, Manassas, VA, USA) are a model for hereditary breast cancer cell line that has a homozygous mutation for the *BRCA1* 5382C terminal; The HCC193 are a model of triple negative breast cancer. The culture medium for HCC1937 was Roswell Park Memorial Institute (RPMI) (ATCC, Manassas, VA, USA) implemented with 20% (*w*/*v*) fetal bovine serum (FBS) (Sigma Aldrich), 100 mg/mL streptomycin, and 100 U/mL penicillin (Sigma Aldrich).

### 2.2. Cells Treatments

#### 2.2.1. H_2_O_2_ Treatment

Cells were seeded until they reached 60% of confluence. Cells were serum-starved for 24 h and subsequently treated with 250 μM H_2_O_2_ (Sigma Aldrich) in complete medium for 24 h.

#### 2.2.2. LY294002 Treatment

Cells were seeded until they reached 60% of confluence. Cells were serum-starved for 24 h and subsequently treated with 30 μM LY294002 in complete medium for 24 h.

#### 2.2.3. H_2_O_2_ and LY294002 Co-Treatment

Cells were seeded until they reached 60% of confluence. Cells were serum-starved for 24 h. Complete medium, containing 30 μM LY294002, was added to the cell plate. Following cells were treated with 250 μM H_2_O_2_ (Sigma Aldrich) for 24 h.

### 2.3. Whole Protein Extraction

Cells were washed with PBS and incubated on ice for 30 min, using lysis buffer containing 15 mM Tris pH 7.5, 25 mM KCl, 120 mM NaCl, and 0.5% Triton X-100 and supplemented with protease and phosphatase inhibitor cocktail (Halt Protease Inhibitor Cocktail/Halt Phosphatase Inhibitor Cocktail, Thermo Fisher Scientific Waltham, MA, USA). Cell lysate was sonicated at 4 °C for 10″ three times. Afterward, it was centrifugated at 15,000× *g* for 30 min. The resulting supernatant was accurately transferred to a new tube. The protein concentration was measured by the Bradford method (Bio-Rad, Hercules, CA, USA) [15]. Proteins extracts were stored at −80 °C until use.

### 2.4. Isolation of Nuclear Fractions

Cells were incubated with lysis buffer containing 10 mM Tris-Cl pH 8.0, 1 mM KCl, 1.5 mM MgCl_2_, and 1 mM DTT, supplemented with protease and phosphatase inhibitor cocktail. The mixture was incubated for 30 min on rotator at 4 °C.

Nuclei were pelleted at 10,000× *g* for 10 min at 4 °C. Protein assay was done using the Bradford Protein Assay (Bio-Rad, Hercules, CA, USA)) according to the manufacturer’s instructions with bovin serum albumin (BSA) as standards [15].

Western blot against Vimentin (5741, Cell signaling, 1:1000) and Histone H3 (1:1000; 9715; Cell Signaling; Danvers, MA, USA) were used to ensure that nuclear extracts were not contaminated by cytoplasmic fraction.

### 2.5. Two-Dimensional Polyacrylamide Gel Electrophoresis (2DE) Analysis

To perform a 2DE, 130μg of cell proteins extract were solubilized using isoelectrofocusing buffer (IEF). The buffer contains 4% CHAPS, 8 M urea, 0.1 M dithiothreitol (DTT), 0.8% pH 3–10 nonlinear (NL) carrier ampholyte buffer. IEF was performed at 70,000 Vh, on the IPGphor II apparatus (GE Healthcare, Chicago, IL, USA), using nonlinear Immobline Dry Strips (GE Healthcare), pH 3–10, 24 cm long. After this first dimension, the strips were equilibrated with SDS equilibration buffer containing DTT 10 mg/mL^−1^, for 15 min and then for another 15 min in SDS equilibration buffer with iodoacetamide (IAA)25 mg/mL^−1^. Procedures were performed according to GE Healthcare Ettan protocol book [16,17]. The second dimension was carried out on 10% SDS polyacrylamide gels, until the bromophenol blue reached the bottom of the gels [18]. The gels were fixed and stained using silver staining method, which is compatible with mass spectrometry analysis [19].

For each sample, the analysis was carried out in triplicate. Gel images were acquired using Image Scanner II (GE Healthcare, Chicago, IL, USA) and analyzed through Image Master 2D-Platinum software 6.0 version (GE Healthcare, Chicago, IL, USA)

### 2.6. In-Gel Digestion

Protein spots were excised from the gel, de-stained, and trypsinized using an in-gel procedure previously reported. For proteins bands from SDS gel, before proceeding with digestion, spots were further reduced and alkylated using, respectively, DTT (10 mg/mL^−1^) and IAA (25 mg/mL^−1^). Tryptic peptides were purified with Pierce C18 Spin Columns (Thermo Fisher Scientific Inc.), eluted with 90 μL of 70% acetonitrile, and dehydrated in a vacuum evaporator [20,21,22]. Mass analysis was carried out using Nanoscale liquid chromatography coupled with tandem mass spectrometry.

### 2.7. Nanoscale LC-MS/MS Analysis

Tandem Mass analysis was carried out with Easy LC 1000 nanoscale liquid chromatography (nanoLC) system (Thermo Fisher Scientific, Odense, Denmark). The chromatography was performed on a C18 silica capillary, 75 μm i.d. The tryptic peptides were injected into the analytical column at 500 nL min^−1^ and then eluted with a binary gradient (Phase A: 0.1% formic acid, 2% acetonitrile; phase B: 0.1% formic acid, 80% acetonitrile; the elution gradient was set up at 350 nL min^−1^ flow rate). For the mass detection, a quadrupole, Orbitrap mass spectrometer Q-Extactive (Thermo Fisher Scientific, Bremen, Germany) coupled with a nano electrospray (nESI) with a potential of 1800 V was used, operating in positive ion mode. MS/MS analysis was done a Data-dependent top-6 method.

The window for precursor ion isolation was set at 2.0 *m*/*z*^−1^ and the collision energy was normalized at 30 s. The dynamic exclusion was set at 15 s and the for-triggering MS/MS events ion threshold was 2 × 10^4^.

For data processing, the software Proteome Discoverer 1.4 (Thermo Fisher Scientific, Bremen, Germany) was used, with Sequest as a search engine, and the Human-refprot-isoforms.fasta as sequence database.

Searching parameters were: 15 ppm of MS tolerance; 0.02 Da MS/MS tolerance; fixed modification: carbamidomethylation of cysteine; variable modification: phosphorylation of serine, tyrosine, and threonine; oxidation of methionine; max. missed cleavages 2; taxonomy Human.

The identification for protein was based on two tryptic peptides hits result with medium confidence (Xcorr > 2.0 for doubly charged peptides, >2.5 for triply charged peptides, and >3.0 for peptides having a charge state >3 to considerate a peptide identification valid) [23]. Peptides carrying MG-H1 modification were considered valid only if the score of identification was high [23].

### 2.8. Western Blot Analysis

Western blot analysis was carried out with precast SDS-PAGE (Any kDTM Mini-PROTEAN Precast Protein Gels, Bio-Rad) and 15% SDS-PAGE. Gels were electrotransferred to a nitrocellulose membrane using a Transblot turbo system (Bio-Rad). Membrane blocking, before primary antibody hybridization, was performed according with antibody manufacturer instructions. Membranes were incubated with following primary antibodies overnight: Akt (1:1000; 9272; Cell Signaling; Danvers, MA, USA); Phospho-Akt (1:1000; 4060; Cell Signaling; Danvers, MA, USA); DJ-1 (1:1000; D29E5XP, Cell Signaling; Danvers, MA, USA); Phospho-Akt Substrate HRP conjugate (1:1000; 6950; Cell Signaling; Danvers, MA, USA); Anti-Methylglyoxal (1:2000; STA-011; Cell Biolabs; San Diego, CA, USA); γ-tubulin (1:1000; sc-7396; Santa Cruz; Dallas, TX, USA); H1 (1:100; ab 71594; Abcam; Cambridge, UK); H3 (1:1000; 9715; Cell Signaling; Danvers, MA, USA); H4 (1:1000; 2592; Cell Signaling; Danvers, MA, USA). Vimentin (5741, Cell signaling, 1:1000).The detection of primary antibody was performed with a horseradish peroxidase-conjugate secondary antibody: antirabbit (1:3000, 7074; Cell Signaling; Danvers, MA, USA); antimouse (1:2000, sc-7074; Santa Cruz, Dallas, TX, USA); antigoat (1:4000; sc-2354; Santa Cruz, Dallas, TX, USA).

Cell signaling antibodies were diluted in 1X TBS, 0.1% Tween-20 with 5% *w*/*v* BSA. Abcam and Santa Cruz antibodies were diluted in 1X TBS, 0.1% Tween-20 with 5% *w*/*v* nonfat dry milk according with manufacturer instructions.

Immunoblots were developed using the SuperSignal West Femto enhanced chemiluminescent substrate (Pierce, Thermo Fisher Scientific Inc., Bremen, Germany). Corresponding images were acquired by Alliance 2.7 (UVITEC, Eppendorf, Milan, Italy). Densitometric analysis was done by Alliance 1D fully automated software.

Data were examined and plotted by means of Excel spreadsheet (Microsoft, Redmond, WA, USA). They were expressed as mean ± SEM (N): SEM is the standard error of the mean and N is the number of experimental repeats.

### 2.9. 2D Western Blot Analysis

20 μg of proteins extract, from breast cancer cells, were diluted in Isoelectrofocusing (IEF) sample buffer, containing: 8 M urea, 0.1 M DTT, 4% CHAPS, 0.8% pH 3–10 carrier ampholyte buffer. IEF was performed on 7-cm-long Immoline DryStrips, pH 3–10 (GE Healthcare). Run was performed on the IPGphor II apparatus (GE Healthcare) until a total of 30,000 Vh was reached. After this first step, IPG strips were equilibrated with SDS equilibration solution that contains 10 mg/mL DTT, and subsequently equilibrated with SDS buffer containing 25 mg/mL IAA.

Proteins were resolved, according to the molecular weight, on 12% SDS-polyacrylamide gels (2 W/gel; 25 °C) until the dye front reached the bottom of the gels. Gels were electrotransferred to a nitrocellulose membrane by a Trans-blot turbo system (Bio-Rad). The membranes were incubated with primary antibody DJ-1 (1:1000; D29E5XP, Cell Signaling; Danvers, MA, USA) O.N. at 4 °C. The detection of primary antibody was performed with a horseradish peroxidase-conjugate secondary anti-rabbit (1:3000, 7074; Cell Signaling; Danvers, MA, USA). Blots were developed using the SuperSignal West Femto ECL substrate (Pierce, Thermo Fisher Scientific Inc., Bremen, Germany). The images of blotted proteins were acquired by Alliance 4.7 (UVITEC, Eppendorf, Milan, Italy), the intensities of blotted proteins it was determined using the densitometric software (Alliance 1D fully automated software).

Image analysis of 2D Western blot was carried out with Image Master 2D Platinum software 6.0 (GE Healthcare). Proteoform levels were quantified using the relative volume (% Vol) option of the software. Each proteoform was quantified respect the whole spot volume of each isoelectric series. This option allows the data to be independent of experimental variations between membrane caused by differences in loading. Differences between sample were expressed as fold change over the baseline. Analysis was performed using three independent experiments, respectively. All data were presented as mean ± SEM (N), where SEM represents the standard error of the mean and N indicates the number of experimental repeats. Data were plotted using Excel spreadsheet (Microsoft Corporation, Redmond, WA, USA).

### 2.10. Scansite Analysis

Scansite is a motif scanning program (https://scansite4.mit.edu/4.0) [24]. It was used to find out putative kinase substrates. The program looks for domain-binding peptides or kinase substrates, applying a position-specific scoring matrix (PSSM) originated from screening peptides libraries synthesized chemically or displayed in bacteriophages.

The search engine was used to analyze phosphorylated peptides and identify the substrates that are likely to be phosphorylated by the basophilic serine/threonine kinase Akt. Putative protein phosphorylation site was further investigated by evaluating evolutionary conservation

### 2.11. Immunofluorescence Microscopy Analysis

First, 55 × 10^5^ cells were plated on glass coverslips in 6-well culture dish. When the cells reached the 50% of confluences, MitoTracker Green (Thermo Scientific) was added to the cell for 45′ at 37 °C. Cells were washed three times with pre-warmed phosphate buffer saline (PBS) and for 30 min with 4% paraformaldehyde (Sigma-Aldrich), Cells permeabilization was done with 0.3% Triton X-100 (Sigma-Aldrich) diluted in PBS for 15 min. Nonspecific-binding sites were blocked with 10% FBS (Biowest, Nauaillé, France) and 0.1% Triton X-100 in PBS for 1 h at RT. The primary antibody against DJ-1 (D29E5XP, Cell Signaling Danvers, MA, USA) was diluted 1:1000 in PBS, 3% FBS.

A solution, with secondary goat anti-rabbit Alexa-Fluor-594 (A-11012; Life Technologies) (2 μg/mL) in PBS containing 1% FBS, and DAPI (4′,6-diamidino-2-phenylindole, to stain the nuclei) was incubated for 45 min at RT. Coverslips were mounted with fluorescent mounting medium (Dako Cytomation). Images were acquired using DMi8 Leica microscope (Leica Microsystem Srl, Milan, Italy). The experiment was repeated in three independent biological replicates.

To ascertain and quantify subcellular localization of expressed DJ-1 protein, the relative staining intensities were assessed in the mitochondria and nucleus. The analysis of the images was carried out with ImageJ software (Wayne Rasband, National Institute of Mental Health, Bethesda, MD, USA). DAPI and MitoTracker staining were used to define the nuclear and mitochondria regions of interest (ROIs) that were drawn manually.

The contrast and brightness were adjusted and images were randomly selected from more 25 cells per cell line. The non-specific staining of the ROIs was manually selected from images to become background in which intensity value was subtracted from the image content. Total cell fluorescence (CTFC) was corrected and evaluated by the formula of Integrated Density: Area of selected cell * Mean fluorescence of background readings. Finally, the average ratio between the intensity of fluorescence in the mitochondria and nuclei was plotted [25].

### 2.12. Histones Acid Extraction

Histone protein extraction was done in acid condition. Cells, at 80% of confluence, were washed with 500 μL of hypotonic lysis buffer (10 mM Tris-HCl pH 8.0, 1 mM KCl, 1.5 mM MgCl_2_, 1 mM DTT) implemented with protease and phosphatase inhibitor cocktail (Halt Protease Inhibitor Cocktail/Halt Phosphatase Inhibitor Cocktail Thermo Fisher Scientific Inc.). Plate was scraped with 1 mL of hypotonic lysis buffer, and incubated for 30 min on rotator at 4 °C. The resulting nuclei were isolated by centrifugation at 4 °C for 10 min at 10,000× *g*. Histones were extracted by incubating the pellet with 0.4 N H_2_SO_4_ on ice. Proteins were precipitated with trichloroacetic acid (TCA). Resulting histone pellet was washed twice with ice-cold acetone, lyophilized, and solubilized in sterile H_2_O. The protein concentration was measured by the Bradford method (Bio-Rad, Hercules, CA, USA) [15]. Histones extracts were stored at −80 °C until use.

### 2.13. 2D Gel Triton-Acid-Urea (TAU) Electrophoresis

#### 2.13.1. First Dimension: Triton-Acid-Urea (TAU) Gel Electrophoresis

TAU gel was cast According to Shechter et al., protocol. Lyophilized histones (35 µg) were solubilized in acidic sample buffer (6 M Urea, 5% glacial acid acetic, 0.02%) and loaded on the gel. It was run in 5% acetic acid solution for 17 h at 25 V. Histones isoforms resolved on the gel were stained with EZBlue Gel Staining Reagent (Sigma Aldrich) [26].

#### 2.13.2. Second Dimension: SDS-PAGE

The histones lines were cut off from the gel and loaded on a 12% SDS polyacrylamide gels (Mini-PROTEAN^®^ TGX^TM^ Precast Gels, IPG Well) to resolve them by molecular weight.

The separation was run at 80 V until the dye reached the bottom of the gel. The gels were stained with staining procedure compatible with mass spectrometry: EZBlue Gel Staining Reagent (Sigma Aldrich) or silver staining. Image analysis of the gels was executed with Image Master 2D-Platinum software 6.0 (GE Healthcare). All analysis was carried out in triplicate [19].

### 2.14. 2D Western Blot TAU

Equal amounts of histone extracts were separated by 2D TAU/SDS gel. In order to circumvent gel to gel variation and reliably compare samples under investigation, second dimension was done on Mini-PROTEAN^®^ TGX™ Precast Gels, IPG Well (12%). Resulting gel was blotted to nitrocellulose membranes with a Trans-blot turbo system (Bio-Rad) using Trans-Blot^®^ Turbo™ Mini Nitrocellulose Transfer Packs. Filters were incubated with the following antibody: DJ-1 (1:1000; Cell Signaling; Danvers, MA, USA) O.N. Equal protein loading was ensured by incubating membranes with red ponceau solution (P7170 Sigma-Aldrich).

The images of blotted proteins were acquired by Alliance 4.7 (UVITEC, Eppendorf, Milan, Italy), the intensities of blotted proteins was determined using the densitometric software Alliance 1D fully automated software. Image analysis of 2D Western blot was carried out with Image Master 2D Platinum software 6.0 (GE Healthcare) [13].

### 2.15. Histones Spot Extractions

Proteins spots on 2D TAU gel was processed according with the protocol reported in Section 2.6, “In-Gel Digestion”.

### 2.16. MS Data Processing and Database Searching

All acquired data, stored by raw data files, were preprocessed with Proteome Discoverer 1.4 (Thermo Fisher Scientific, Bremen, Germany). MS/MS data were sought on the Human UniProt database. To estimate the false discovery rate (FDR) of peptide identifications it was utilizing the “Target-decoy PSM validator” node in Proteome Discoverer. Searching parameters were MS error tolerance: 5 ppm; MS/MS error tolerance: 0.02 Da; enzyme specificity: trypsin; maximum number of missed cleavages: 2; taxonomy Human; fixed modifications: Carbamidomethylation (C); variable modification: Oxidation (M), Acetyl (K), Methyl (K), Dimethyl (K), Trimethyl (K), Methyl (R), Dimethyl (R), Deamidated (R), Phosphorylation (STY), and Glycation (MG-H1). The identification for protein was based on two tryptic peptides hits result with high confidence (Xcorr > 2.0 for doubly charged peptides, >2.5 for triply charged peptides, and >3.0 for peptides having a charge state >3 to considerate a peptide identification valid) [27].

### 2.17. Immunoprecipitation Analysis

Next, 100 µg of proteins from whole proteins extract, was incubated overnight with Phospho-Akt Substrate Sepharose^®^ Bead Conjugate (1:20; 9646; Cell Signaling; Danvers, MA, USA) to perform an immunoprecipitation. Beads were washed three times and were incubated at 100 °C for 5 min. The resultant immunocomplexes were resolved through precast SDS-PAGE (Any kDTM Mini-PROTEAN Precast Protein Gels, Bio-Rad); proteins were electrotransferred by a Transblot turbo system (Bio-Rad) for 30 min according to Bio-Rad protocol. Membrane was incubated with primary antibody: DJ-1 (1:1000; D29E5XP, Cell Signaling; Danvers, MA, USA). The detection of primary antibody was executed with antirabbit horseradish peroxidase conjugate secondary antibody (1:3000; Cell Signaling; Danvers; MA, USA). With SuperSignal West Femto ECL substrate (Pierce, Thermo Fisher Scientific Inc., Bremen, Germany); and images of blotted antibodies were acquired by Alliance 2.7 (UVITEC, Eppendorf, Milan, Italy). γ-Tubulin was used as loading control.

### 2.18. Immunoprecipitation Isolation of DJ-1 Nuclear Interactor

100 µg of nuclear extract, isolated as previously reported, were incubated with the antibody against DJ-1 (1:100; D29E5XP, Cell Signaling; Danvers, MA, USA) at 4 °C for 4 h on a rotating device. 20 µL of resuspended volume of Protein A/G PLUS-Agarose (Santa Cruz) were added to the mixture and incubated at 4 °C on a rotating device overnight. Beads were washed three times and were incubated at 100 °C for 5 min. In parallel, a Mock-IP was done using only Protein A/G PLUS-Agarose, as negative control. The resultant immunocomplexes and the input were resolved through precast SDS-PAGE (Any kDTM Mini-PROTEAN Precast Protein Gels, Bio-Rad); proteins were electrotransferred by a Transblot turbo system (Bio-Rad) for 30 min according to Bio-Rad protocol. Membrane was incubated with primary antibody: DJ-1 (1:1000; D29E5XP, Cell Signaling; Danvers, MA, USA); H3 (1:1000; 9715; Cell Signaling; Danvers, MA, USA); Histone H2B (D2H6). The detection of primary antibody was performed with a horseradish peroxidase-conjugate secondary antibody: antirabbit (1:3000, 7074; Cell Signaling; Danvers, MA, USA); antimouse (1:2000, sc-7074; Santa Cruz, Dallas, TX, USA); and antigoat (1:4000; sc-2354; Santa Cruz, Dallas, TX, USA). Antibodies were diluted according to manufacturer instructions.

Immunoblots were developed using the SuperSignal West Femto ECL substrate (Pierce, Thermo Fisher Scientific Inc., Bremen, Germany). Corresponding images were acquired by Alliance 2.7 (UVITEC, Eppendorf, Milan, Italy). Densitometric analysis was done by Alliance 1D fully automated software.

### 2.19. LC-MS/MS Analysis of DJ-1 Nuclear Interactors

Nuclear DJ-1 interactors were pull down according with the immunoprecipitation proteins previously reported. DJ-1 interactors were resolved by SDS-PAGE (Any kDTM Mini-PROTEAN Precast Protein Gels, Bio-Rad) and visualized using EZBlue Gel Staining Reagent (Sigma Aldrich). Gel lane referred to DJ-1-IP and Mock-IP were sliced according to molecular weight. Gel bands were processed according to the protocol reported in the Section 2.6.

Proteins identified in the Mock-IP gel line were classified as unspecific interactors and eliminated from the list of specific DJ-1-nuclear interactors.

### 2.20. Molecular Modeling Analysis

Starting from the crystal structure of DJ-1, deposited in the Protein Data Bank with the PDB code 4RKW [28], our molecular modeling studies were performed. By using the DJ-1 PDB model as template, we generated the DJ-1 protein phosphorylated at the position 125 by means of the Maestro tool [29]. For our modeling studies, we used the DJ-1 non-phosphorylated and the DJ-1 phosphorylated at the position 125 models. The two receptor structures were prepared through Protein Preparation Wizard implemented in Maestro, using OLPS-2005 as force-field [30]. Residual crystallographic buffer components were removed, missing side chains were built using the Prime module, hydrogen atoms were added, and side chains protonation states at pH 7.4 were assigned [31,32].

After the preparation, both the models were submitted to 200 ns of Molecular Dynamics simulations (MDs) using Desmond-v5.3 at 300 K temperature and ensemble NPT class [33,34]. The systems were immersed in an orthorhombic box of TIP3P water molecules, extending at least 10 Å from the protein, and counter ions were added to neutralize the system charge.

The resulting trajectories were clustered with respect to Root Mean Square Deviation (RMSD), in order to explore all the collection structures obtained, getting ten representative structures (five for the non-phosphorylated DJ-1 protein and five for the phosphorylated DJ-1 protein). Then, by using the Prime calculate Energy tool [32], we selected, for the further analysis, the *lowest-energy* structure, respectively, for the non-phosphorylated and the phosphorylated protein.

### 2.21. Evaluation of Extracellular Acidification Rates

Extracellular acidification rate (ECAR) measurements were performed using the XFp Extracellular Flux analyzer (Seahorse Bioscience, North Billerica, MA, USA). The analysis was done using the Seahorse XF Cell Energy Phenotype Test kit according with manufacturer instruction. Briefly, cells were plated into XFp polystyrene cell culture plates (Seahorse Bioscience, North Billerica). MCF7 and HCC1937 cells were seeded at 30,000/well (XFp plate). The cells were incubated for 24 h in a humidified 37 °C incubator with DMEM medium or RMPI1640, respectively. Each experiment was done in triplicate according with manufacturer protocol.

## 3. Results

### 3.1. Breast Cancer Cells Use Glycolysis as Principal Source of Energy and Produce AGEs on Critical Histone Residues

**Figure 1:**
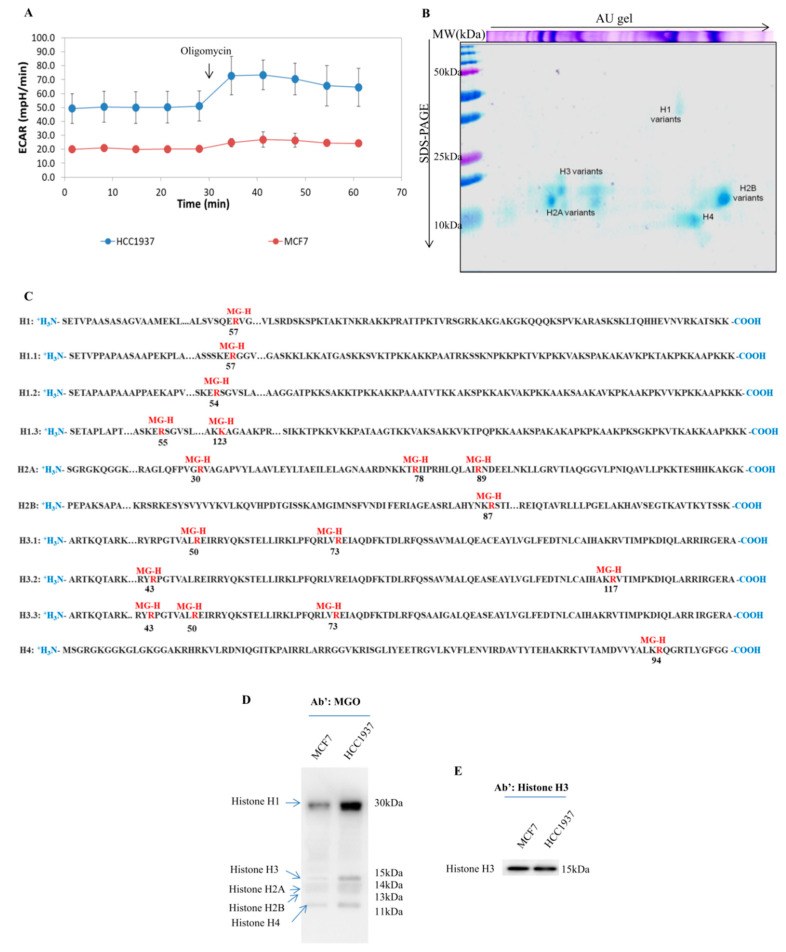
Advanced glycation end-products (AGEs) detection on histone from breast cancer cells; (**A**) Glycolytic activity was measured by the extracellular acidification rate (ECAR). The study was done using the XFp Analyzer. The analysis reveals increased glycolytic activity in HCC1937 breast cancer cells as compared to MCF7 breast cancer cells. The complete report analysis was provided as Supplementary File S1; (**B**) Histones proteins were extracted in acidic conditions and analyzed using Triton-Acid-Urea (TAU) gel. The method resolves very basic proteins combining two perpendicular separation methods. In the first-dimension histones migrate in function of their isoelectric point an hydrophobic properties, in the second dimension histones are resolved based on molecular weight. Histone spots were excised, trypsin digested and identified by LC-MS/MS analysis. Mass spectrometry analysis was done focusing on methylglyoxal (MGO) as post-translational modifications (PTM); (**C**) Mass spectrometry analysis of MGO on histones sequences. In the Figure, the main glycated residue on histone sequences is reported. Each identified glycated residue is bold red. MG-H is acronym of methylglyoxal-derived hydroimidazolone. The number under the red residue indicates the position of the amino acid. LC-MS/MS identification data and MS/MS spectra were provided as Supplementary Files S2 and S3; (**D**) Western blot analysis of MGO-modified histones. Histones proteins were resolved on 15% SDS-PAGE and transferred to a nitrocellulose membrane using the Trans-blot turbo system (Bio-Rad). Membranes were hybridized with primary antibodies against MGO-adducts. Western blot patterns were analyzed using Image Master 2D Platinum software. The analysis allows us to conclude that MGO adducts were more abundant in HCC1937 cells compared to MCF7 breast cancer cells; (**E**) MGO Western blot signal was normalized against the whole levels of Histone H3.

Metabolic rewiring is a key hallmark of cancer and breast cancer cells exhibit a clear shift toward glycolytic metabolism. As shown by ECAR analysis (Figure 1A), this phenomenon considerable in MCF7 cell line, a model of sporadic breast cancer, becomes dramatic in HCC1937, a model of triple-negative breast cancer. This is as expected for cells having to tackle a particularly hostile environment [35,36].

Glycolytic flux induces waste compounds as glyoxals and especially methylglyoxal (Warburg effect) [37] a highly reactive dicarbonyl molecule, responsible, through a non-enzymatic reaction, for the formation of AGEs. Methylglyoxal (MGO) acts principally on lysine, arginine, and cysteine-rich proteins and being highly diffusible enters the nucleus and modifies histones [38].

We mapped AGEs on histones coupling 2D TAU gel with mass spectrometry analysis [13]. Histones proteins extracted in acid conditions were resolved by TAU gel in a single gel. Figure 1B shows a representative 2D TAU histones pattern from MCF7 breast cancer cells. Histone spots marked on the analytic blue gel were excised, de-stained, and in-gel trypsinized.

Mass spectrometry analysis was performed either in MCF7 than in HCC197 breast cancer cells. Proteome discovery software was used for the analysis of MS/MS data. The outputs shaped up a list of histones identifications provided as Supplementary File S2. MS/MS spectra are provided as Supplementary File S3. An overview of histones’ AGEs identifications is shown in Figure 1C. We identified 17 Glycated sites, 16 on arginine and 1 in lysine. The majority of glycation sites identified are positioned on the PFam domains and are located in functional domains. Notably, R57 on histone H1 and R89 on histone H2A were reported as a crucial for methylation and phosphorylation cross-talk [39]. R30 on H2A represents the main target of Protein Arginine Methyltransferase 6 (PRMT6) and then critical in the PRMT6-mediated transcriptional repression [40]. R87 on H2B is determinant for the correct assembly of the H2A-H2B dimer [41]. R54 on H1.2 is a strategic site of ubiquitination [42] and in close proximity to S56, a key site of phosphorylation in breast cancer [43]. R57 on H1.1 was identified as a site of citrullination, located within the DNA-binding site of H1, whose single PTM allows global chromatin decondensation [44]. R78 on H2A id determinant for H2A-H2B recognition and nucleosome editing [45]. The majority of the sites undergoing glycation are implicated in histones interactions that lead to the correct assembly of nucleosome.

Thus, our findings enforce the notion that glycation, inducing histones code deconstruction, represents a crucial factor in aging, as well as in the development of degenerative pathologies.

Being the formations of AGEs a non-enzymatic chemical modification, the glycated adducts should be directly related to the quantity of the reactants (MGO) and to the exposure time. In our breast cancer models Western blot analysis on histones isoforms using anti-MGO antibody clearly shows that all histones isoforms, including H1, undergo glycation (Figure 1D) with the maximum extent in HCC1937 cells coherently with ECAR analysis (Figure 1A).

### 3.2. Identification of a Novel Phosphorylation on DJ-1 Threonine 125, Within a Putative Consensus of Akt

**Figure 2:**
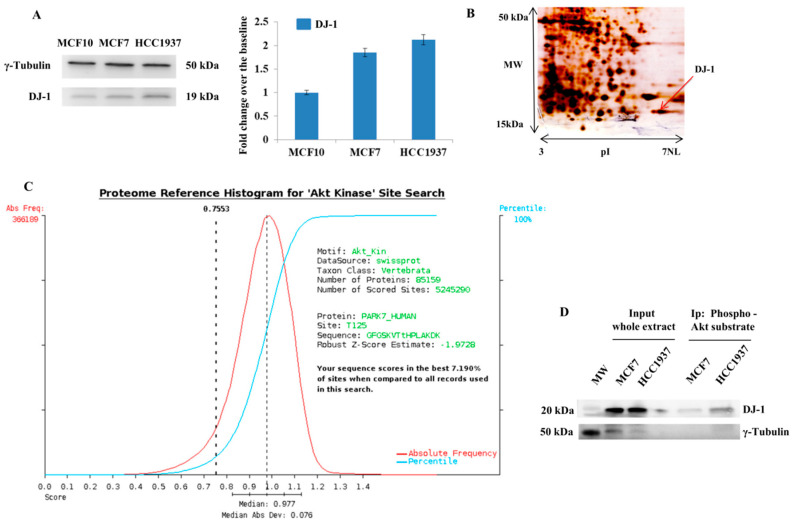
Identification of Novel Phosphorylation on DJ-1 (**A**) Western blot and densitometry analysis of DJ-1 in whole protein extracts from MCF10, MCF7, and HCC1937 cells. Whole protein extracts were resolved using AnykD precast gels and blotted on nitrocellulose membrane using turbo blot. Filter were hybridized with primary antibody against DJ-1 (D29E5XP, Cell Signaling; Danvers, MA, USA). Equal loading of proteins was confirmed using Anti-γ-Tubulin antibody (C-20, Santa Cruz). Each assay was repeated in three independent biological replicates. The histogram shows the level of DJ-1, expressed as fold change over the baseline, in breast cancer cells (MCF7 and HCC1937) compared to normal immortalized breast cells (MCF10); (**B**) 2D gel map of MCF7 breast cancer cells. Protein spot identified as DJ-1, through LC-MS/MS analysis, is indicated with a black arrow; (**C**) scansite results. The scansite search on DJ-1 allows us to detect that the Phospho-peptide VTtHPLAK, identified by LC-MS/MS analysis, encloses a putative Akt consensus; (**D**) The ability of Akt to directly interact with DJ-1 was investigated by immunoprecipitation experiments. We incubated the protein extracts from MCF7 and HCC1937 breast cancer cells using Sepharose Bead Conjugate with Phospho-Akt Substrate (RXXS*/T*) antibody. The input and the immunocomplexes, containing isolated Akt substrates, were resolved by SDS gel and blotted onto nitrocellulose membrane. The membrane was assayed with antibodies against DJ-1. γ-Tubulin was used as loading control.

In cancer cells, the main deglycating agent is DJ-1 [38] an antioxidant enzyme able to catalyze the removal of MGO from histones proteins. Comparing breast cancer cells with normal immortalized counterpart, we found that DJ-1 is overexpressed in tumor cells, especially in HCC1937 cells (Figure 2A).

Analyzing DJ-1 by 2DE we found that, it appears as a train of spots (Figure 2B), laying for the existence of specific proteoforms. Subsequent LC-MS/MS identification (Table 1) allowed us to identify, for the first time to our knowledge, a peculiar DJ-1 phosphorylation (threonine 125). MS/MS identifications is provided in Table 1 and Table 3. The in-silico tool scansite 4 (http://scansite4.mit.edu/) searches for motifs within proteins that are likely to be phosphorylated by specific protein kinases. The search of consensus on DJ-1 enclosing phosphorylated threonine identifies a putative consensus of Akt (Figure 2C). The phylogenic analysis, on this consensus, reports it as evolutionarily conserved (Supplementary File S4).

The ability of Akt1 to directly interact with DJ-1 was investigated by immunoprecipitation experiments. We incubated the protein extract from breast cancer cells using Sepharose Bead Conjugate with Phospho-Akt Substrate (RXXS*/T*) antibody. Western blot analysis on immunoprecipitated Akt substrates clearly confirmed that DJ-1 owns an Akt consensus (Figure 2D).

### 3.3. DJ-1 Proteoforms Are Modulated by Akt Pathway

**Figure 3:**
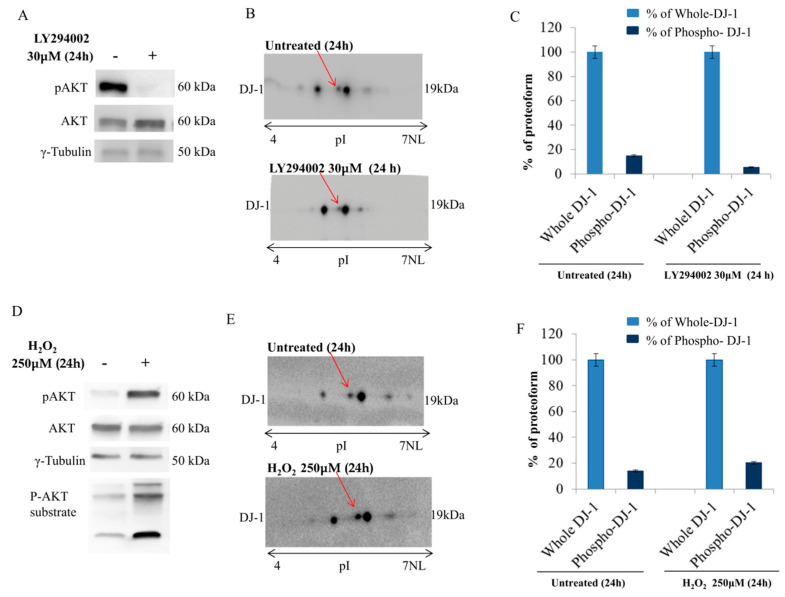
DJ-1 proteoforms analysis in MCF7 breast cancer cells (**A**)Treatment of MCF7 cells with LY294002 (30 μM for 24 h) induces the inhibition of pAkt. Total Akt and γ-tubulin were used as loading control; (**B**) 2D Western blot showing all DJ-1 proteoforms in whole cell extract during LY294002 treatment. LY294002 treatment induces a significant shift of DJ-1 proteoforms (red arrow) toward basic pH according to the loss of acidic proteoforms (**B**); (**C**) Densitometric analysis of DJ-1 proteoform modulated by LY294002 treatment; (**D**) In MCF7 breast cancer cells, mild oxidative stress (H_2_O_2_ 250 µM for 24 h) increases the phosphorylation of Akt together with the phosphorylation of Akt substrates. Total Akt and γ-tubulin were used as loading control; (**E**) 2D Western blot showing all DJ-1 proteoforms in whole cell extract during H_2_O_2_ treatment. During the activation of Akt pathway, it is possible to observe in DJ-1 proteoforms (red arrow) a shift toward acid pH. The shift is coherent with the addition of an acid group to DJ-1; (**F**) Densitometric analysis of DJ-1 proteoform modulated by H_2_O_2_ treatment.

To better assess the ability of Akt to modulate DJ-1 proteoforms we treated breast cancer cells with LY294002, a specific PI3K inhibitor (Figure 3A). DJ-1 proteoforms were analyzed through 2D Western blot analysis. As shown in Figure 3B, the treatment induced a significant shift of DJ-1 proteoforms toward basic pH according with the loss of acidic group. Conversely, the activation of Akt pathway (Figure 3D), treating cancer cells with H_2_O_2_ [46], induced a significant shift toward acid pH according with the gain of an acidic group (Figure 3E). This finding is consistent with an Akt induced modulation of DJ-1 proteoforms.

Densitometry analysis of DJ-1 proteoforms assessed that under standard condition, Phospho-DJ-1 was about 14% of whole DJ-1. After LY294002 (30 μM) treatment, the level of Phospho-DJ-1 significantly decreases (5.6%). Conversely, upon H_2_O_2_ (250 μM) treatment, we observed that the level of Phospho DJ-1 increases to 20.3% concomitantly with Akt phosphorylation (Figure 3C,F).

### 3.4. Activation of Akt Pathway is Crucial for DJ-1 Nuclear Localization

**Figure 4:**
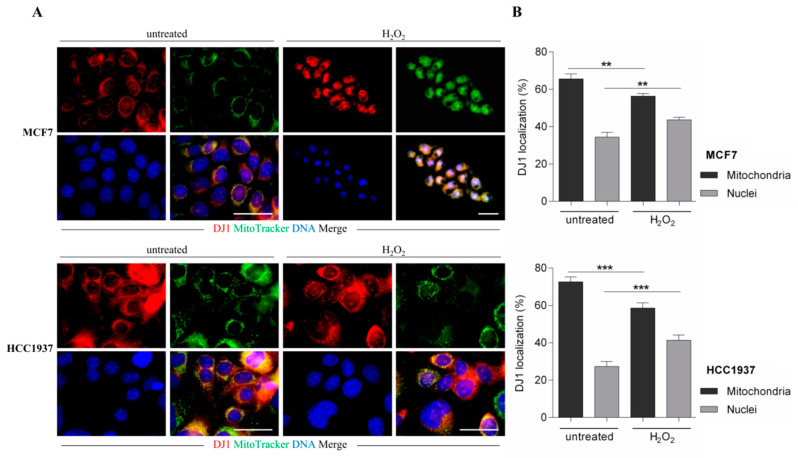
DJ-1 localization in MCF7 and HCC1937 cell line. (**A**) Representative immunofluorescence of DJ-1 (red) in MCF7 and HCC1937 cell lines. Nuclei were counterstained with 4′,6-diamidino-2-phenylindole (DAPI) (blue) and mitochondria were labeled with MitoTracker (green). Scale bar, 50 μm; (**B**) DJ-1 localization analysis performed on mitochondrial and nuclear areas, respectively. Data are mean ± SEM, *n* > 30 cells per condition. ** *p* value ≤ 0.01, *** *p* value ≤ 0.001 (*t*-test).

DJ-1 is a mitochondrial protein that explicates its antioxidant activity in the cytoplasm. As deglycating activity is performed principally in the nucleus, we investigated if the activation of Akt pathway influences DJ-1 subcellular localization. Immunofluorescence analysis on breast cancer cells (MCF7 and HCC1937) assessed that, under standard condition, DJ-1 protein is mostly localized in the mitochondria (65% and 72.7%) than in the nuclei (34.4% and 27.3%) in MCF7 and HCC1937 lines, respectively. Upon H_2_O_2_ (250 μM) treatment, a significant translocation to the nuclei was detected in MCF7 and HCC1937 cell lines, concomitantly with Akt phosphorylation (Figure 4A,B).

### 3.5. Nuclear DJ-1 Directly Interacts with Nucleosome

**Figure 5:**
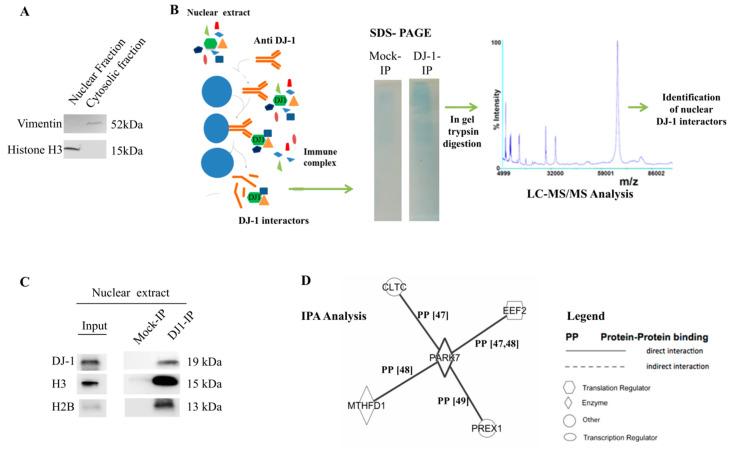
Mass spectrometry identification of DJ-1 nuclear interactors. (**A**) The quality of nuclear extracts was assessed by Western blot using vimentin as marker of cytosolic fraction and Histone H3 as marker of nuclear fraction; (**B**) DJ-1 nuclear interactors were obtained by pull-down nuclear proteins extracted from breast cancer cell (MCF7), using anti-DJ-1 antibody. In parallel, a Mock IP using only protein A/G resin was done to identify unspecific interactors. Immunocomplexes were resolved by SDS-PAGE (Any kD Mini-PROTEAN TGX Precast Protein Gels). Gel bands from mock-IP and DJ-1-IP were manually excised, trypsin digested, and analyzed by LC-MS/MS. Proteins identified in mock-IP were classified as unspecific interactors and eliminated from the list of specific DJ-1 nuclear interactors. The complete list of DJ-1 interactors was provided as Supplementary File S5; (**C**) The interaction of DJ-1 with Histones H2B and H3 was also confirmed by Western blot analysis. Mock-IP was loaded as negative control; (**D**) DJ-1 interactors were analyzed using the ingenuity pathways analysis (IPA) tool. Identified networks were provided as Supplementary File S5. IPA analysis allows us to exploit that some of the identified proteins have just been reported as DJ-1 interactors. The network in panel E confirms the direct interaction of DJ-1 with Clathrin heavy chain 1(CLH1), methylenetetrahydrofolate dehydrogenase (MTHFD1), Elongation factor 2 (EF2), and Phosphatidylinositol 3,4,5-trisphosphate-dependent Rac exchanger 1 protein (PREX1). PP on the line connecting proteins indicates protein-protein binding. Peculiar proteins interactions are provided as Supplementary File S5.

The activity of DJ-1 in the nuclei was investigated coupling an immunoprecipitation (IP) experiment with LC-MS/MS analysis. Anti-DJ-1 antibodies were used to pull down nuclear DJ-1 interactors. They were resolved by SDS-PAGE and analyzed by mass spectrometry (Figure 5B). About 25 DJ-1 interactors were identified and reported as Supplementary File S5. The most intriguing result was the detection of histones isoforms (Table 2). The direct interaction of histones H3 and Histones H2B with DJ-1 was further confirmed by Western blot analysis (Figure 5C). Finally, we analyzed DJ-1-interactors using ingenuity pathways analysis (IPA) that mapped identified proteins onto two main networks: in which associated diseases and functions were transcriptional modification, cancer, and cell death and survival (Supplementary File S5). Interestingly, we established that, among identified proteins, four were just well-known direct DJ-1 interactors with a nuclear localization (Figure 5D) [47,48,49].

### 3.6. Phosphorylated DJ-1 Proteoform Localizes with Nucleosome

**Figure 6:**
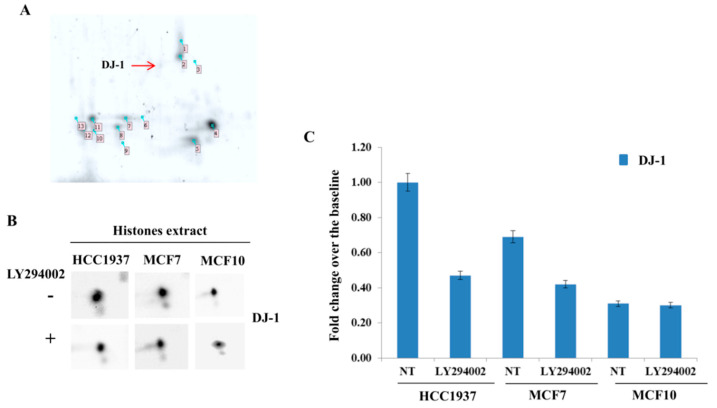
Two-dimensional TAU Gel and Western blot of DJ-1 interacting with histones. (**A**) TAU gel map of histone isoforms (HCC1937). Histones were resolved coupling TAU gel and SDS-PAGE. Numbered histones spots were extracted and analyzed by mass spectrometry. Experiments are representative of three biologic replicates. Histones protein identifications are reported as Supplementary File S2. The red arrow indicates DJ-1 protein spot that associates with histones; (**B**) 2D TAU Western blot analysis lets to focus that DJ-1 proteoform interacting with histones appears as single spot. The expression levels of DJ-1 associated with histones are much higher in HCC1937 compared to MCF7 breast cancer cells. However, the levels of DJ-1 associated with histone was lower in normal immortalized breast cells (MCF10) compared to breast cancer cells (HCC1937 and MCF7). The assay was repeated in three independent biological replicates; Blot signal was acquired using Alliance 2.7 (UVITEC, Eppendorf, Milan, Italy). Membranes were acquired at 6 s. Treatment with LY294002 (30 μM) decreases the levels of DJ-1 interacting with histones both in HCC1937 and MCF7 breast cancer cells. Relative densitometry analyses are shown in panel (**C**). Images relative to western signal normalization were provided as Supplementary File S6.

As we hypothesized that the DJ-1 proteoforms directly interacts with nucleosome when phosphorylated, we isolated nucleosome and resolved histones using 2D TAU gel. On the 2D TAU map, it was possible to visualize some proteins spot with a peculiar migration pattern not attributable to histones (Figure 6A). Using mass spectrometry analysis, we identified those proteins and one of them was DJ-1. The in-depth analysis of identified peptides allows us to conclude that the spot was referred to the phosphorylated DJ-1 proteoform (Table 3). Western blot analysis on the histones’ extracts, resolved by 2D TAU gel, clearly confirmed the presence of DJ-1, pointing out that DJ-1 associated with histones appears as a single spot. Interestingly, the level of DJ-1 associated with histone was much higher HCC1937 compared to MFC7 breast cancer cells (Figure 6B,C). Based on these results, we conclude that the only proteoform of DJ-1 associating with histones is the phosphorylated one.

To enforce the finding, we performed 2D tau Western blot on nucleosome of cells treated with the Akt inhibitor LY294002. Akt inhibition induced a significant decrease of PhosphoDJ-1 associated with nucleosomes (Figure 6B,C).

### 3.7. Effects of Threonine 125 Phosphorylation on DJ-1 Structure and Function

As shown above we found a novel phosphorylation on DJ-1 at Thr125. Analyzing the DJ-1 3D structure, we observed that the residue Thr125 is adjacent to His126, a key residue of DJ-1 catalytic site. Glyoxalase activity of DJ-1 is attributable to a catalytic pocket including Glu18, His126, and Cys106 and that works similarly to the proteolytic triade of Cysteine protease [50].

**Figure 7:**
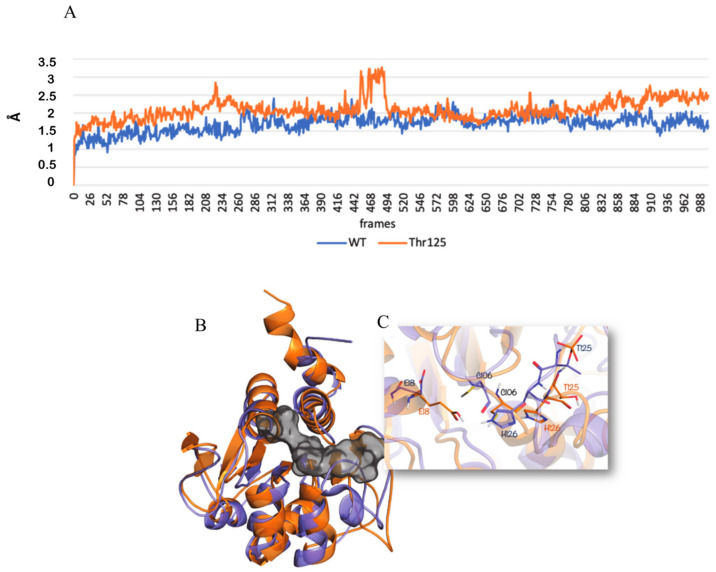
(**A**) Root Mean Square Deviation (RMSD) trend of non-phosphorylated DJ-1 (blue line) and phosphorylated DJ-1 at position 125 (orange line); 3D representation of (**B**) DJ-1 protein non-phosphorylated and DJ-1 protein phosphorylated overlapped, shown as orange and slate cartoon, respectively. The catalytic binding site is represented as a gray surface; (**C**) DJ-1 catalytic binding site, the residues are shown as orange and slate sticks for the non-phosphorylated and phosphorylated DJ-1, respectively.

In order to analyze the effect of Thr125 phosphorylation on the DJ-1 protein stability, in silico analysis were carried out. Molecular dynamics simulations results highlighted a reduced DJ-1 protein stability in the presence of phosphorylated threonine 125, with respect to the non-phosphorylated DJ-1 (Figure 7A).

Being the threonine 125 close to the histidine 126, one of the three residues of the catalytic triade (Glu18, Cys106, His126) of the DJ-1 active site, with the aim to evaluate the effect of the phosphorylation on the catalytic site, we applied a Visual Inspection analysis on the two selected DJ-1 protein structures. We noticed that, in the presence of the Thr125 phosphorylation, there is a conformational change of the catalytic residues triade (Figure 7B,C). Thus, Thr125 phosphorylation seems to induce a change in DJ-1 conformation modulating its catalytic activity. This could justify the altered activity of phosphorylated DJ-1.

### 3.8. Akt Pathway Modulates DJ-1 Glyoxalase Activity

During metabolic switching to glycolysis, levels of waste product as MGO increases concomitantly with the AGE products on histone proteins that ultimately might induce cell death. Cancer cells need to counteract AGE formation to promote cell survival. DJ-1 phosphorylation might be part of this mechanism to evade cell death.

To confirm our hypothesis, we treated cancer cells with H_2_O_2_ in presence and in the absence of LY294002. Akt phosphorylation and AGE modified histones level were analyzed by Western blot.

As expected, cells treated with LY294002 showed, in comparison with non-inhibited cells, a reduced level of Phospho-Akt even when it was been induced by H_2_O_2_. Akt inhibition was always followed by reduced levels of nuclear DJ-1 and increased MGO on histones proteins.

**Figure 8:**
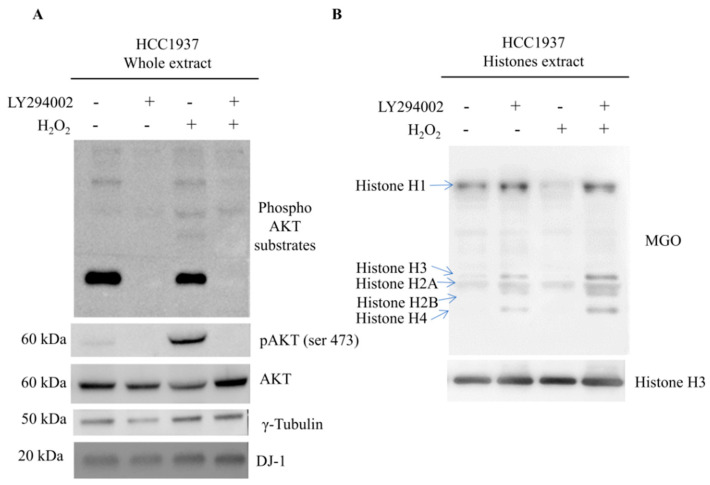
Western blot analysis relative to the expression of Akt activation and AGEs formations in HCC1937 breast cancer cells (**A**) Western blot analysis on whole extract from HCC1937. Levels of Phospho-Akt substrates, Phospho-Akt and DJ-1 are shown in loading control and treated cells. Signals of Phospho antibody were normalized against the total level of Akt. A goat polyclonal anti-γ-Tubulin antibody was used (C-20) to confirm equal loading of whole proteins. Proteins were resolved using Any kD Mini-PROTEAN TGX Precast Protein Gels and blotted using trans-blot turbo system (Bio-Rad). Signals intensities were assessed by densitometry using the Alliance 4.7; (**B**) Western blot analysis on histones extract from HCC1937. Levels of MGO are shown in loading control and treated cells for each histone isoforms resolved on the gel. The major level of MGO are detected on Histone H1. Histones were resolved using 15% Mini-PROTEAN TGX Precast Protein Gels and blotted using trans-blot turbo system (Bio-Rad). Signals intensities were assessed by densitometry using the Alliance 4.7.

**Figure 9:**
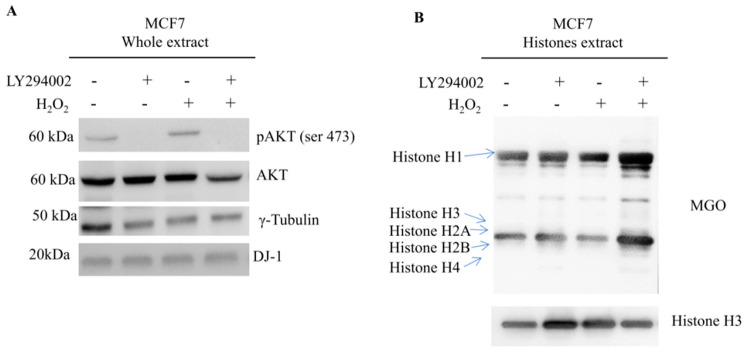
Western blot analysis relative to the expression of Akt activation and AGEs formations in MCF7 breast cancer cells (**A**) Western blot analysis on whole extract from MCF7. Levels of Phospho-Akt and DJ-1 are shown in loading control and treated cells. Signals of Phospho antibody were normalized against the total level of Akt. A goat polyclonal anti-γ-Tubulin antibody (C-20) was used to confirm equal loading of whole proteins. Proteins were resolved using Any kD Mini-PROTEAN TGX Precast Protein Gels and blotted using trans-blot turbo system (Bio-Rad). Signals intensities were assessed by densitometry using the Alliance 4.7 software; (**B**) Western blot analysis on histones extracts from MCF7. Levels of MGO are shown in loading control and treated cells for each histone isoforms resolved on the gel. The major level of MGO are detected on Histone H1. Histones were resolved using 15% Mini-PROTEAN TGX Precast Protein Gels and blotted using trans-blot turbo system (Bio-Rad). Signals intensities were assessed by densitometry using the Alliance 4.7 software.

Experiments were carried out in HCC1937 breast cancer cells (Figure 8) and in MCF7 breast cancer cells (Figure 9). This experiment allows us to conclude that Akt induced DJ-1 phosphorylation is crucial for glyoxalase activity.

## 4. Discussion

In this work, we aimed to elucidate the strategies that breast cancer cells exploit to overcome the formation of AGE on histones following the metabolic rewiring toward aerobic glycolysis. Cancer cells largely rely on glycolysis to produce energy needed for cellular economy even during normoxia conditions. ‘Warburg effect’ is the main hallmark of cancer cells. The increased glycolytic flux induces carbonyl stress, through the overproduction of harmful metabolites as MGO. MGO is able to bypass nuclear membrane and reacts with amino groups of lysine and arginine of histone tails leading to the formation of advanced glycation end products (AGEs). This reaction underlies the loss of “Histone Code” [51].

Here, the formation of AGEs on histones was investigated applying a powerful proteomic approach. The methods, previously reported by us for the global analysis of histones posttranslational modification [13], enables us to resolve all histones isoforms in a single 2D map, allowing us to investigate all histones PTMs, even those less represented. The strategies allow us to disclose several AGEs modification on histone proteins, including, for the first time to our knowledge, those on histone H1.

Recently, some authors investigated the formation of AGE on histones addressing the issue in several cellular models, including breast cancer, but mostly forcing MGO concentrations and/or the Glycolytic flux [38,51].

As the AGEs formation is a non-enzymatic reaction, induced variations of glycolytic flux and/or increase of MGO may significantly affect the magnitude of results obtained, making them not immediately related to what really happens in breast cancers cells.

Although the findings reported represent an excellent starting point for our work, we chose of not interfere with the delicate metabolism balance of our cellular models, keeping “endogenous conditions”. Our strategies allow us to identify several MGO adducts on histones proteins, firstly the formation of MGO adducts on the histones H1. This novel modification, detected on arginine 57, was never reported by others. The histone H1, also known as the linker histone, has the function to strongly compact the nucleosomes, acting as strategic planners of chromatin structure [52].

Several finding reports that histone H1 is a key factor both in chromatin condensation and decondensation, the presence of a bulky modification, as MGO, on R57 should be relevant for H1 activity due to its close proximity to R54, a residue crucial for chromatin disassembly [53].

The overall analysis of sites undergoing glycation reveals that all them have been related to the crosstalk between phosphorylation and acetylation. It enforces the hypothesis that AGEs formation might act in the deconstruction of histone code either directly modifying sites undergoing PTMs, either inducing steric impediments when modifying sites close to critical amino acids.

Mass spectrometry analysis of different breast cancer cell lines enabled the identification of several histone sites undergoing MGO adducts but without any specificity in terms of residues involved. However, by Western blot, quantitative differences became clear, being the amount of AGEs significantly higher in HCC1937 cells, a model of triple negative breast cancer (TNBC), therefore, with a direct correlation with glycolytic flux extent.

Several findings report DJ-1 as the main glyoxalase responsible for AGEs removal from histones, but they lack clarification of how this novel function integrates with the well-known DJ-1 antioxidant activity.

DJ-1 is functionally elusive, and it adds up protection of both normal and cancer cells from oxidative stress acting in the cytoplasm with the efficient removal of AGEs in the nucleus.

We provided here evidence of multiple DJ-1 proteoforms accountable for its different functions. We disclosed a novel proteoform that, according to our result, might be specifically capable of nuclear localization and AGEs removal from histones. Coupling 2D gel with mass spectrometry, we identified a novel phosphorylation on DJ-1 (threonine 125) in which expression level correlates with activation of a mitogenic pathway. The in-silico analysis of the DJ-1 sequence allows location of this phosphorylation within a consensus recognizable as putative substrates of Akt and immunoprecipitation experiments enforced the hypothesis of a DJ-1/Akt interaction. In addition, modulation of the Akt pathway correlates with DJ-1 nuclear localization and influences its proteoforms, inducing the loss and/or the gain of an acidic group in function of its inactivation or activation, respectively. In addition, a single DJ-1 proteoform seems able to interact with histones as DJ-1 associated with nucleosome appears in the 2D map as a single spot, accounting for the hypothesis that only Phospho-DJ-1 associates with histones. Once again, a modulation of the Akt pathway correlates with levels of phospho-DJ-1 associated with the nucleosome.

The novel site of phosphorylation is the threonine 125. It is adjacent to histidine 126 and then very close to the catalytic triad of the glyoxalase enzymatic site. The activity of DJ-1 glyoxalase implies three key amino acids, Glu18, Cys106, and His126, working in a catalytic pocket similarly to a cysteine protease. Taking into account literature data [54] the antioxidant and the glyoxalase activity seems not coexisting but largely related to compartmentalization and to the cellular functional status. It is implausible that DJ-1 acts widely as a glyoxalase since it should have a constitutive pro-survival role, indiscriminately working as a cysteine protease in every one cellular compartment.

Much more likely, phosphorylation on thr125 might account for nuclear localization and activation of DJ-1 glyoxalase by inducing a molecular rearrangement of the active site. On the catalytic site, thr125 phosphorylation elicits a conformational change that destabilizes the enzymatic pocket and increases the reactivity of the molecules. Data we presented here, although not conclusive, strongly enforce that hypothesis. The enhancement of enzymatic activity is supported by the finding that in cells showing high levels of Phospho-DJ-1, a decrease of MGO adducts on histones isoforms has been detected, as well. Indeed, AGEs on histones isoforms, increase after Akt inhibition by LY294002 treatment. It means that the inhibition of the Akt does not allow the activation of DJ-1 glyoxalase, very likely because of lack of threonine phosphorylation.

Our data propose a model in which DJ-1 might have a dual role. It is ubiquitously expressed and acts as a redox-sensitive chaperone and as a sensor for oxidative stress. Upon activation of the Akt pathway, it undergoes phosphorylation, translocates into the nucleus where DJ-1 is detectable only as phosphorylated proteoform, and becomes an active glyoxalase removing from histones the AGEs, formed because of augmented glycolytic flux. This mechanism preserving histones code favors malignant cells integrity and sustains survival.

## 5. Conclusions

This work provides new insights into DJ-1 proteoforms and how they might act within the program that cancer cells execute to escape aging and preserve survival.

The formations of AGEs products on histones proteins are associated with degenerative pathologies mostly by the deconstruction of histones code that leads to early aging and, ultimately, cells death.

The switching of cancer cells to glycolysis increasing MGO concentrations and then AGEs should be a pro-death event. However, in cancer cells, especially breast cancer cells, rewiring their metabolism increases aggressiveness and improves their capability to survive in very hard conditions.

In this scenario, the elucidation of mechanism that allows cancer cells to counteract AGEs formation, primarily the deglycating activity of DJ-1, might be determinant for defining novel therapeutical approaches. Considering the central role that DJ-1 fulfills, as antioxidant agents, ubiquitously expressed, its direct inhibition might result in a backfire. The mapping of DJ-1 proteoforms and their functional characterization is crucial for the definition of novel molecular targets. Our work gives a substantial contribution to the hypothesis of precisely target deglycase activity of DJ-1 preserving its antioxidant activity.

Overall, we depicted a strategy that cancer cells elicit to overcome aging and preserve their immortality, disclosing a novel therapeutic target and pointing out to the notion that the modulation of specific DJ-1 function might produce substantial anticancer effects.

## Figures and Tables

**Table 1:** DJ-1 LC-MS/MS Identification. Protein spots from gel shown in Figure 2B, were excised, trypsin digested, and identified by LC-MS/MS analysis. In the table below, DJ-1 identification is reported. The phosphorylated peptide in threonine is underlined in red. In the table are: Accession number, Univocal code identifying the protein on the Uniprot database (https://www.uniprot.org); Description of identified protein; Score of identification; Coverage, percentage of amino acid identified by MS/MS analysis; Peptides, number of peptides uniquely identifying the protein; PSMs, total number of identified peptide spectra matched for the protein; AAs, MW, molecular weight of identified protein; pI, theoretical isoelectric point of identified protein; Sequence of identified peptide; PSMs, total number of identified peptide spectra matched for the peptides; Modification, position of modified residue and type of modification; MH+ Molecular weight of positive ion; and Miss cleavages, number of allowed missed cleavage. White row is referred to protein MS data. Blue rows are referred to peptide MS data.

Accession	Description	Score	Coverage	Peptides	PSMs	MW (kDa)	pI
Q99497	Protein DJ-1	20.85	41.27	9	18	19.9	6.79
	**Sequence**		**PSMs**	**Modifications**		**MH+ (Da)**	**Mis Cleav.**
**Peptides**	DVVIcPDASLEDAKK		1	C5(Carbamidomethyl)		1659.8	1
	GAEEmETVIPVDVMR		1	M5(Oxidation)		1691.8	0
	GAEEmETVIPVDVmR		1	M5(Oxidation); M14(Oxidation)		1707.7	0
	VTVAGLAGK		2			815.5	0
	EILKEQENR		2			1158.6	1
	VTTHPLAK		1			866.5	0
	DGLILTSR		3			874.5	0
	ALVILAK		2			727.5	0
	APLVLKD		2			755.4	1
	VTtHPLAK		2	T3(Phospho)		946.4	0
	EILK		1			502.3	0

**Table 2:** DJ-1-interactors of particular interest. In the table are reported: Accession number, Univocal code identifying the protein on the Uniprot database (https://www.uniprot.org); Protein description; Score for protein identification; PSMs, total number of identified peptide spectra matched for the protein; MW, molecular weight of identified protein; pI, theoretical isoelectric point of identified protein. The complete list of interactors is provided as Supplementary File S5.

Accession	Description	Score	Unique Peptides	PSMs	AAs	MW (kDa)	pI
P62805	Histone H4 [H4_HUMAN]	37.68	3	13	103	11.4	11.36
O60814	Histone H2B [H2B1K_HUMAN]	13.67	3	5	126	13.9	10.32
P68431	Histone H3. [H31_HUMAN]	49.55	2	21	136	15.4	11.12

**Table 3:** Mass spectrometry analysis confirms that DJ-1, interacting with histones, carries a peculiar phosphorylation on Threonine included in the Akt consensus. In the table are: Accession number, Univocal code identifying the protein on the Uniprot database (https://www.uniprot.org); Description of identified protein; Coverage, percentage of amino acid identified by MS/MS analysis; Unique peptides, number of peptides uniquely identifying the protein; PSMs, total number of identified peptide spectra matched for the protein; Score of identification; MW, molecular weight of identified protein; Calc pI, theoretical isoelectric point of identified protein; Sequence of identified peptide; PSMs, total number of identified peptide spectra matched for the peptide; Modification, position of modified residue and type of modification; MH+ Molecular weight of positive ion; Miss cleavage, number of allowed missed cleavages. White row is referred to protein MS data. Blue rows are referred to peptide MS data.

Accession	Description	Coverage	Unique Peptides	PSMs	Score	MW (kDa)	pI
Q99497	Protein DJ-1	64.55	11	58	102.40	19.9	6.79
	**Sequence**	**PSMs**	**Modifications**		**MH+ (Da)**	**Mis Cleav.**	
Peptides	EGPYDVVVLPGGNLGAQNLSESAAVK	2			2584.3	0	
	VTVAGLAGKDPVQCSR	6	C14(Carbamidomethyl)		1657.8	1	
	VTVAGLAGKDPVQCSR	2	C14(Carbamidomethyl); R16(Methyl)		1671.8	1	
	DKMMNGGHYTYSENR	3	M3(Oxidation); M4(Oxidation)		1834.7	1	
	DVVICPDASLEDAKK	6	C5(Carbamidomethyl)		1659.8	1	
	GAEEMETVIPVDVMR	6	M5(Oxidation); M14(Oxidation)		1707.7	0	
	VTVAGLAGKDPVQCSR	1	C14(Carbamidomethyl); R16(Deamidated)		1658.8	1	
	VEKDGLILTSR	2			1230.7	1	
	MMNGGHYTYSENR	2	M1(Oxidation); M2(Oxidation)		1591.6	0	
	VTTHPLAK	8			866.5	0	
	VTVAGLAGK	4			815.5	0	
	DGLILTSR	5			874.5	0	
	EILKEQENR	6			1158.6	1	
	ALVILAK	1			727.5	0	
	VTTHPLAK	2	T3(Phospho)		946.4	0	

## Supplementary Materials

The following are available online at https://www.mdpi.com/2073-4409/9/9/1968/s1: Supplementary File S1: Seahorse XF Cell Energy Phenotype Test. The analysis was carried out on HCC1937 and MCF7 breast cancer cells; Supplementary File S2: In the table are summarized the results of the LC-MS/MS identifications. For each identification, we reported the number by which spot are marked on Figure 1, the accession number, the number of identified peptides and the % of sequence coverage. Mass spectrometry data are averages of three biologic replicates; Supplementary File S3: Fragmentation MS/MS spectra of the modified peptide carrying identified PTMs. MS/MS data are referred to all analyzed histones. Data were analyzed by Proteome Discoverer 1.4 software. MS/MS data were searched on the Human UniProt database; Supplementary File S4: The phylogenic analysis for the consensus VTTHPLAK; Supplementary File S5: (A) List of DJ-1-interactors: In the table are summarized the results of the LC-MS/MS identifications. For each identification, we reported the accession number, the number of identified peptides and the % of sequence coverage. Mass spectrometry data are averages of three biologic replicates; (B)Uncropped image of Blue gel used for LC-MS/MS analysis; (C) IPA analysis of DJ-1-interactors; (D) Ipa network of direct DJ-1 nuclear interactors; Supplementary File S6: Proteins normalization for 2D TAU Western blot shown in Figure 6.
