# Supplementary material for: DJ-1 Proteoforms in Breast Cancer Cells: The Escape of Metabolic Epigenetic Misregulation
Source: Cells. 2020 Aug 26;9(9):1968. doi: 10.3390/cells9091968 (PMC7563694; doi:10.3390/cells9091968)
Supplement: Supplementary file 1 [file cells-09-01968-s001.zip › SI_03/Supplemtary file 3.pdf]

MS/MS SPECTRA

Sequence: ERGGVSLAALKK, R2-MG-H1 (54.01057 Da)
Charge: +3, Monoisotopic m/z: 428.25430 Da (+0.17 mmu/+0.4 ppm), MH+: 1282.74836 Da,
RT: 12.35 min,
Identified with: Sequest HT (v1.3); XCorr:2.72, Ions matched by search engine: 0/0
Fragment match tolerance used for search: 0.02 Da
Fragments used for search: a; a-H_2O; a-NH_3; b; b-H_2O; b-NH_3; y; y-H_2O; y-NH_3
Protein references (1):
- Histone H1.1 OS=Homo sapiens GN=HIST1H1A PE=1 SV=3 - [H11_HUMAN]

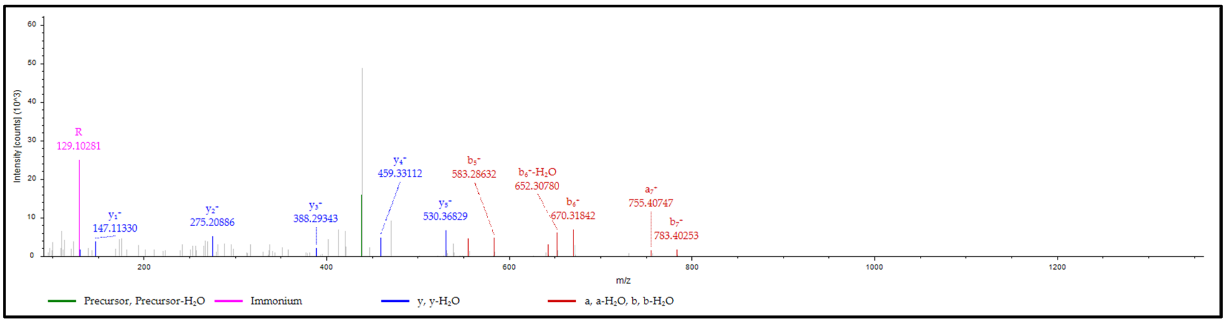

#1	Immonium	a^+	a^{2+}	a^{3+}	b^+	b^{2+}	b^{3+}	Seq.	y^+	y^{2+}	y^{3+}	#2
1	102.05496	102.05496	51.53112	34.68984	130.04988	65.52858	44.02148	E				12
2	129.11348	312.16665	156.58696	104.72707	340.16156	170.58442	114.05870	R-MG-H1	1183.71580	592.36154	395.24345	11
3	60.04439	399.19868	200.10298	133.73774	427.19359	214.10043	143.06938	S	973.60411	487.30569	325.20622	10
4	30.03383	456.22015	228.61371	152.74490	484.21506	242.61117	162.07654	G	886.57208	443.78968	296.19554	9
5	72.08078	555.28857	278.14792	185.76771	583.28348	292.14538	195.09934	V	829.55061	415.27894	277.18839	8
6	60.04439	642.32060	321.66394	214.77838	670.31551	335.66139	224.11002	S	730.48219	365.74473	244.16558	7
7	86.09643	755.40467	378.20597	252.47307	783.39958	392.20343	261.80471	L	643.45016	322.22872	215.15490	6
8	44.04948	826.44179	413.72453	276.15211	854.43670	427.72199	285.48375	A	530.36609	265.68668	177.46021	5
9	44.04948	897.47891	449.24309	299.83115	925.47382	463.24055	309.16279	A	459.32897	230.16812	153.78117	4
10	86.09643	1010.56298	505.78513	337.52584	1038.55789	519.78258	346.85748	L	388.29185	194.64956	130.10213	3
11	101.10733	1138.65795	569.83261	380.22417	1166.65286	583.83007	389.55580	K	275.20778	138.10753	92.40744	2
12								K	147.11281	74.06004	49.70912	1

Sequence: ERGGVSLAALK, R2-MG-H1 (54.01057 Da)

Charge: +2,Monoisotopic m/z: 577.83276 Da (+2.69 mmu/+4.66 ppm), MH+: 1154.65825 Da, RT: 14.85 min,
Identified with: Sequest HT (v1.3); XCorr:2.54, Ions matched by search engine: 0/0
Fragment match tolerance used for search: 0.02 Da
Fragments used for search: a; a-H_2O; a-NH_3; b; b-H_2O; b-NH_3; y; y-H_2O; y-NH_3
Protein references (1):
Histone H1.1 OS=Homo sapiens GN=HIST1H1A PE=1 SV=3 - [H11_HUMAN]

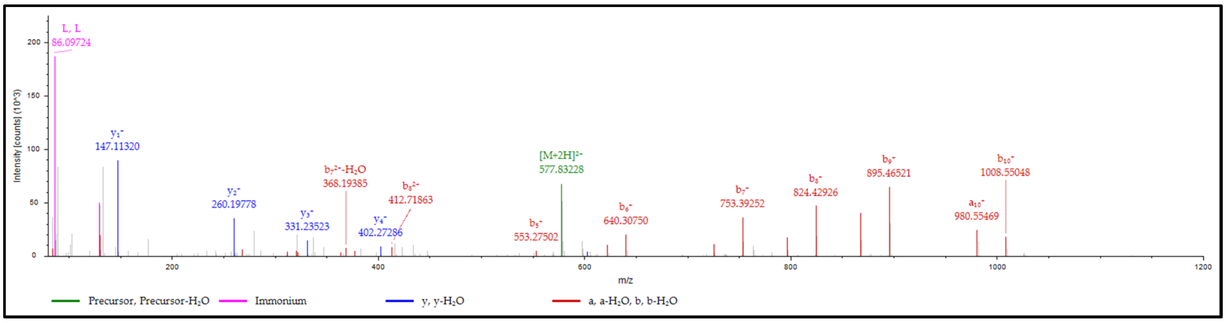

#1	Immonium	a⁺	a²⁺	b⁺	b²⁺	Seq.	y⁺	y²⁺	#2
1	102.05496	102.05496	51.53112	130.04988	65.52858	E			11
2	129.11348	312.16665	156.58696	340.16156	170.58442	R-MG-H1	1025.61027	513.30877	10
3	30.03383	369.18812	185.09770	397.18303	199.09515	G	815.49858	408.25293	9
4	30.03383	426.20959	213.60843	454.20450	227.60589	G	758.47711	379.74219	8
5	72.08078	525.27801	263.14264	553.27292	277.14010	V	701.45564	351.23146	7
6	60.04439	612.31004	306.65866	640.30495	320.65611	S	602.38722	301.69725	6
7	86.09643	725.39411	363.20069	753.38902	377.19815	L	515.35519	258.18123	5
8	44.04948	796.43123	398.71925	824.42614	412.71671	A	402.27112	201.63920	4
9	44.04948	867.46835	434.23781	895.46326	448.23527	A	331.23400	166.12064	3
10	86.09643	980.55242	490.77985	1008.54733	504.77730	L	260.19688	130.60208	2
11						K	147.11281	74.06004	1

Sequence: AGLQFPVGR, R9-MG-H1 (54.01057 Da)
Charge: +2,   Monoisotopic m/z: 499.77615 Da (+1.59 mmu/+3.19 ppm),   MH+: 998.54503 Da,   RT: 14.70 min,
Identified with: Sequest HT (v1.3); XCorr:2.08, Ions matched by search engine: 0/0
Fragment match tolerance used for search: 0.02 Da
Fragments used for search: a; a-NH_3; b; b-NH_3; y; y-NH_3
Protein references (15):
- Histone H2A type 1-A OS=Homo sapiens GN=HIST1H2AA PE=1 SV=3 - [H2A1A_HUMAN]
- Histone H2A type 1-B/E OS=Homo sapiens GN=HIST1H2AB PE=1 SV=2 - [H2A1B_HUMAN]
- Histone H2A type 1-C OS=Homo sapiens GN=HIST1H2AC PE=1 SV=3 - [H2A1C_HUMAN]
- Histone H2A type 1-D OS=Homo sapiens GN=HIST1H2AD PE=1 SV=2 - [H2A1D_HUMAN]
- Histone H2A type 1-H OS=Homo sapiens GN=HIST1H2AH PE=1 SV=3 - [H2A1H_HUMAN]
- Histone H2A type 1-J OS=Homo sapiens GN=HIST1H2AJ PE=1 SV=3 - [H2A1J_HUMAN]
- Histone H2A type 1 OS=Homo sapiens GN=HIST1H2AG PE=1 SV=2 - [H2A1_HUMAN]
- Histone H2A type 2-A OS=Homo sapiens GN=HIST2H2AA3 PE=1 SV=3 - [H2A2A_HUMAN]
- Histone H2A type 2-B OS=Homo sapiens GN=HIST2H2AB PE=1 SV=3 - [H2A2B_HUMAN]
- Histone H2A type 2-C OS=Homo sapiens GN=HIST2H2AC PE=1 SV=4 - [H2A2C_HUMAN]
- Histone H2A type 3 OS=Homo sapiens GN=HIST3H2A PE=1 SV=3 - [H2A3_HUMAN]
- Histone H2A.J OS=Homo sapiens GN=H2AFJ PE=1 SV=1 - [H2AJ_HUMAN]
- Histone H2A.V OS=Homo sapiens GN=H2AFV PE=1 SV=3 - [H2AV_HUMAN]
- Histone H2AX OS=Homo sapiens GN=H2AFX PE=1 SV=2 - [H2AX_HUMAN]

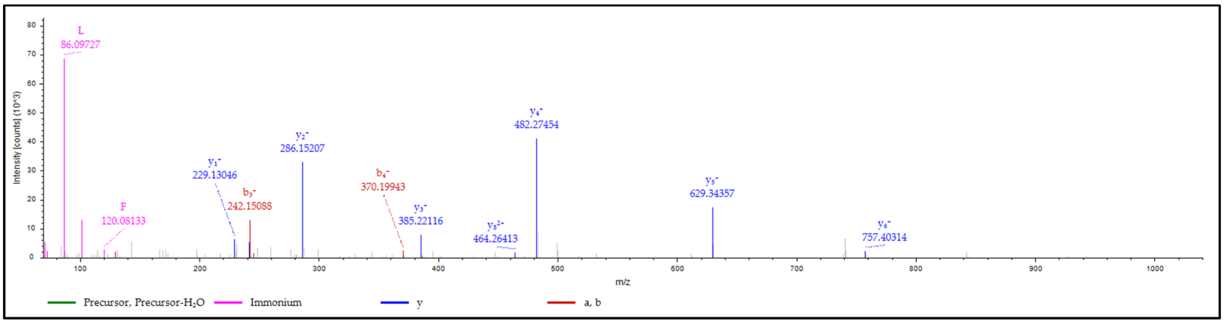

#1	Immonium	a⁺	a²⁺	b⁺	b²⁺	Seq.	y⁺	y²⁺	#2
1	44.04948	44.04948	22.52838	72.04440	36.52584	A			9
2	30.03383	101.07095	51.03911	129.06587	65.03657	G	927.50473	464.25600	8
3	86.09643	214.15502	107.58115	242.14994	121.57861	L	870.48326	435.74527	7
4	101.07094	342.21360	171.61044	370.20852	185.60790	Q	757.39919	379.20323	6
5	120.08078	489.28202	245.14465	517.27694	259.14211	F	629.34061	315.17394	5
6	70.06513	586.33479	293.67103	614.32971	307.66849	P	482.27219	241.63973	4
7	72.08078	685.40321	343.20524	713.39813	357.20270	V	385.21942	193.11335	3
8	30.03383	742.42468	371.71598	770.41960	385.71344	G	286.15100	143.57914	2
9						R-MG-H1	229.12953	115.06840	1

Sequence: HLQLAIR, R7-MG-H1 (54.01057 Da)
Charge: +2, Monoisotopic m/z: 452.77289 Da (+1.08 mmu/+2.38 ppm), MH+: 904.53850 Da, RT: 11.45 min,
Identified with: Sequest HT (v1.3); XCorr:2.18, Ions matched by search engine: 0/0
Fragment match tolerance used for search: 0.02 Da
Fragments used for search: a; a-NH_3; b; b-NH_3; y; y-NH_3
Protein references (14):
- Histone H2A type 1-A OS=Homo sapiens GN=HIST1H2AA PE=1 SV=3 - [H2A1A_HUMAN]
- Histone H2A type 1-B/E OS=Homo sapiens GN=HIST1H2AB PE=1 SV=2 - [H2A1B_HUMAN]
- Histone H2A type 1-C OS=Homo sapiens GN=HIST1H2AC PE=1 SV=3 - [H2A1C_HUMAN]
- Histone H2A type 1-D OS=Homo sapiens GN=HIST1H2AD PE=1 SV=2 - [H2A1D_HUMAN]
- Histone H2A type 1-H OS=Homo sapiens GN=HIST1H2AH PE=1 SV=3 - [H2A1H_HUMAN]
- Histone H2A type 1-J OS=Homo sapiens GN=HIST1H2AJ PE=1 SV=3 - [H2A1J_HUMAN]
- Histone H2A type 1 OS=Homo sapiens GN=HIST1H2AG PE=1 SV=2 - [H2A1_HUMAN]
- Histone H2A type 2-A OS=Homo sapiens GN=HIST2H2AA3 PE=1 SV=3 - [H2A2A_HUMAN]
- Histone H2A type 2-C OS=Homo sapiens GN=HIST2H2AC PE=1 SV=4 - [H2A2C_HUMAN]
- Histone H2A type 3 OS=Homo sapiens GN=HIST3H2A PE=1 SV=3 - [H2A3_HUMAN]
- Histone H2A.J OS=Homo sapiens GN=H2AFJ PE=1 SV=1 - [H2AJ_HUMAN]
- Histone H2A.V OS=Homo sapiens GN=H2AFV PE=1 SV=3 - [H2AV_HUMAN]
- Histone H2AX OS=Homo sapiens GN=H2AFX PE=1 SV=2 - [H2AX_HUMAN]
- Histone H2A.Z OS=Homo sapiens GN=H2AFZ PE=1 SV=2 - [H2AZ_HUMAN]

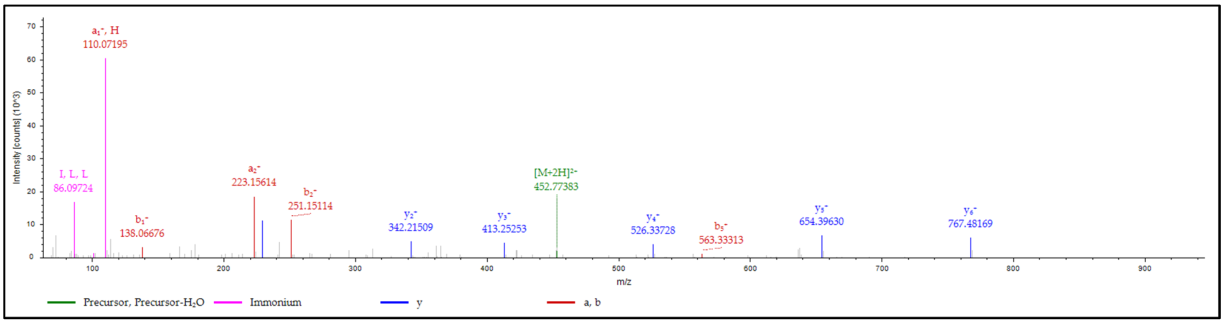

#1	Immonium	a^+	a^{2+}	b^+	b^{2+}	Seq.	y^+	y^{2+}	#2
1	110.07127	110.07127	55.53927	138.06619	69.53673	H			7
2	86.09643	223.15534	112.08131	251.15026	126.07877	L	767.47744	384.24236	6
3	101.07094	351.21392	176.11060	379.20884	190.10806	Q	654.39337	327.70032	5
4	86.09643	464.29799	232.65263	492.29291	246.65009	L	526.33479	263.67103	4
5	44.04948	535.33511	268.17119	563.33003	282.16865	A	413.25072	207.12900	3
6	86.09643	648.41918	324.71323	676.41410	338.71069	I	342.21360	171.61044	2
7						R-MG-H1	229.12953	115.06840	1

Sequence: RSTITSR, R1-MG-H1 (54.01057 Da)
Charge: +2,   Monoisotopic m/z: 437.74393 Da (+3.22 mmu/+7.37 ppm),   MH+: 874.48058 Da,   RT: 4.35 min,
Identified with: Sequest HT (v1.3); XCorr:2.49, Ions matched by search engine: 0/0
Fragment match tolerance used for search: 0.02 Da
Fragments used for search: a; a-H_2O; a-NH_3; b; b-H_2O; b-NH_3; y; y-H_2O; y-NH_3
Protein references (16):
- Histone H2B type 1-B OS=Homo sapiens GN=HIST1H2BB PE=1 SV=2 - [H2B1B_HUMAN]
- Histone H2B type 1-C/E/F/G/I OS=Homo sapiens GN=HIST1H2BC PE=1 SV=4 - [H2B1C_HUMAN]
- Histone H2B type 1-D OS=Homo sapiens GN=HIST1H2BD PE=1 SV=2 - [H2B1D_HUMAN]
- Histone H2B type 1-H OS=Homo sapiens GN=HIST1H2BH PE=1 SV=3 - [H2B1H_HUMAN]
- Histone H2B type 1-J OS=Homo sapiens GN=HIST1H2BJ PE=1 SV=3 - [H2B1J_HUMAN]
- Histone H2B type 1-K OS=Homo sapiens GN=HIST1H2BK PE=1 SV=3 - [H2B1K_HUMAN]
- Histone H2B type 1-L OS=Homo sapiens GN=HIST1H2BL PE=1 SV=3 - [H2B1L_HUMAN]
- Histone H2B type 1-M OS=Homo sapiens GN=HIST1H2BM PE=1 SV=3 - [H2B1M_HUMAN]
- Histone H2B type 1-N OS=Homo sapiens GN=HIST1H2BN PE=1 SV=3 - [H2B1N_HUMAN]
- Histone H2B type 1-O OS=Homo sapiens GN=HIST1H2BO PE=1 SV=3 - [H2B1O_HUMAN]
- Histone H2B type 2-F OS=Homo sapiens GN=HIST2H2BF PE=1 SV=3 - [H2B2F_HUMAN]
-

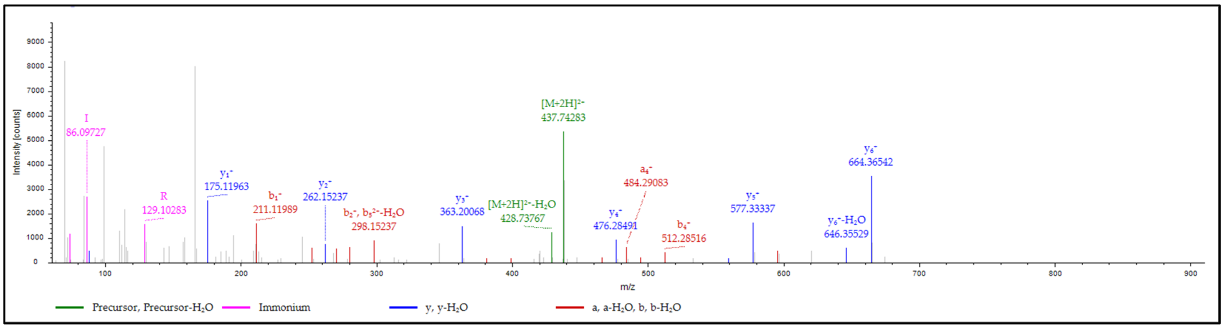

#1	Immonium	a^+	a^{2+}	b^+	b^{2+}	Seq.	y^+	y^{2+}	#2
1	129.11348	183.12405	92.06566	211.11896	106.06312	R-MG-H1			7
2	60.04439	270.15608	135.58168	298.15099	149.57913	S	664.36245	332.68486	6
3	74.06004	371.20376	186.10552	399.19867	200.10297	T	577.33042	289.16885	5
4	86.09643	484.28783	242.64755	512.28274	256.64501	I	476.28274	238.64501	4
5	74.06004	585.33551	293.17139	613.33042	307.16885	T	363.19867	182.10297	3
6	60.04439	672.36754	336.68741	700.36245	350.68486	S	262.15099	131.57913	2
7						R	175.11896	88.06312	1

Sequence: YRPGTVALR, R2-MG-H1 (54.01057 Da)
Charge: +3, Monoisotopic m/z: 362.87457 Da (+1.23 mmu/+3.38 ppm), MH+: 1086.60917 Da, RT: 9.05 min,
Identified with: Sequest HT (v1.3); XCorr:2.34, Ions matched by search engine: 0/0
Fragment match tolerance used for search: 0.02 Da
Fragments used for search: a; a-H_2O; a-NH_3; b; b-H_2O; b-NH_3; y; y-H_2O; y-NH_3
Protein references (5):
- Histone H3.1t OS=Homo sapiens GN=HIST3H3 PE=1 SV=3 - [H31T_HUMAN]
- Histone H3.1 OS=Homo sapiens GN=HIST1H3A PE=1 SV=2 - [H31_HUMAN]
- Histone H3.2 OS=Homo sapiens GN=HIST2H3A PE=1 SV=3 - [H32_HUMAN]
- Histone H3.3 OS=Homo sapiens GN=H3F3A PE=1 SV=2 - [H33_HUMAN]
- Histone H3.3C OS=Homo sapiens GN=H3F3C PE=1 SV=3 - [H3C_HUMAN]

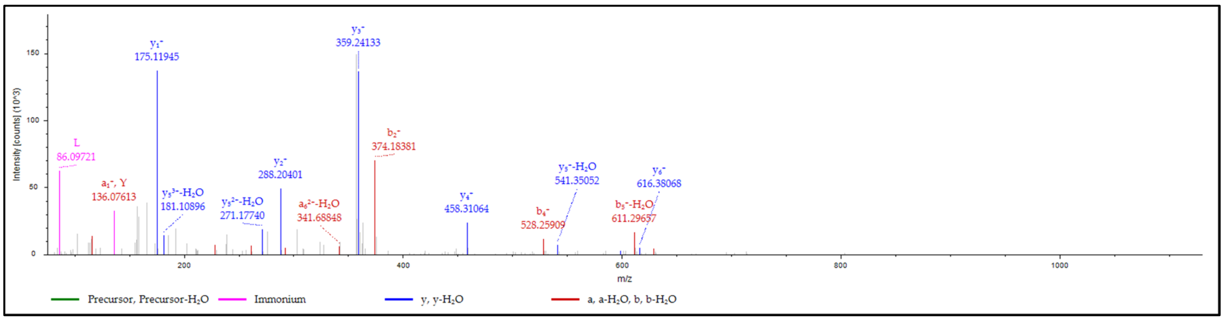

#1	Immonium	a⁺	a²⁺	a³⁺	b⁺	b²⁺	b³⁺	Seq.	y⁺	y²⁺	y³⁺	#2
1	136.07568	136.07568	68.54148	46.03008	164.07060	82.53894	55.36172	Y				9
2	129.11348	346.18737	173.59732	116.06731	374.18228	187.59478	125.39894	R-MG-H1	923.54218	462.27473	308.51891	8
3	70.06513	443.24014	222.12371	148.41823	471.23505	236.12116	157.74987	P	713.43049	357.21888	238.48168	7
4	30.03383	500.26161	250.63444	167.42539	528.25652	264.63190	176.75702	G	616.37772	308.69250	206.13076	6
5	74.06004	601.30929	301.15828	201.10795	629.30420	315.15574	210.43958	T	559.35625	280.18176	187.12360	5
6	72.08078	700.37771	350.69249	234.13075	728.37262	364.68995	243.46239	V	458.30857	229.65792	153.44104	4
7	44.04948	771.41483	386.21105	257.80979	799.40974	400.20851	267.14143	A	359.24015	180.12371	120.41823	3
8	86.09643	884.49890	442.75309	295.50448	912.49381	456.75054	304.83612	L	288.20303	144.60515	96.73919	2
9								R	175.11896	88.06312	59.04450	1

Sequence: RVTIMPK, R1-MG-H1 (54.01057 Da), M5-Oxidation (15.99492 Da)
Charge: +2, Monoisotopic m/z: 457.76285 Da (+2.79 mmu/+6.09 ppm), MH+: 914.51842 Da, RT: 6.07 min,
Identified with: Sequest HT (v1.3); XCorr:2.15, Ions matched by search engine: 0/0
Fragment match tolerance used for search: 0.02 Da
Fragments used for search: a; a-H_2O; a-NH_3; b; b-H_2O; b-NH_3; y; y-H_2O; y-NH_3
Protein references (5):
- Histone H3.1t OS=Homo sapiens GN=HIST3H3 PE=1 SV=3 - [H31T_HUMAN]
- Histone H3.1 OS=Homo sapiens GN=HIST1H3A PE=1 SV=2 - [H31_HUMAN]
- Histone H3.2 OS=Homo sapiens GN=HIST2H3A PE=1 SV=3 - [H32_HUMAN]
- Histone H3.3 OS=Homo sapiens GN=H3F3A PE=1 SV=2 - [H33_HUMAN]
- Histone H3.3C OS=Homo sapiens GN=H3F3C PE=1 SV=3 - [H3C_HUMAN]

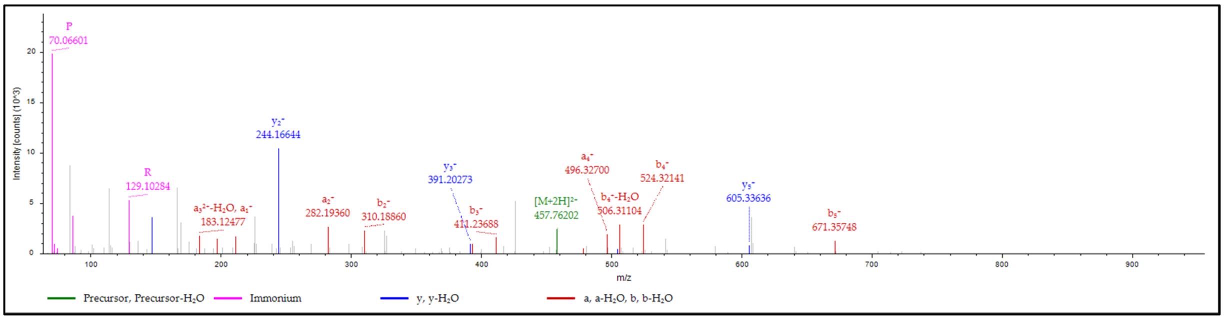

#1	Immonium	a⁺	a²⁺	b⁺	b²⁺	Seq.	y⁺	y²⁺	#2
1	129.11348	183.12405	92.06566	211.11896	106.06312	R-MG-H1			7
2	72.08078	282.19247	141.59987	310.18738	155.59733	V	704.40117	352.70422	6
3	74.06004	383.24015	192.12371	411.23506	206.12117	T	605.33275	303.17001	5
4	86.09643	496.32422	248.66575	524.31913	262.66320	I	504.28507	252.64617	4
5	104.05286	643.35963	322.18345	671.35455	336.18091	M-Oxidation	391.20100	196.10414	3
6	70.06513	740.41240	370.70984	768.40732	384.70730	P	244.16558	122.58643	2
7						K	147.11281	74.06004	1

Sequence: YRPGTVALR, R2-MG-H1 (54.01057 Da)
Charge: +3, Monoisotopic m/z: 362.87558 Da (+2.23 mmu/+6.16 ppm), MH+: 1086.61219 Da, RT: 8.87 min,
Identified with: Sequest HT (v1.3); XCorr:2.38, Ions matched by search engine: 0/0
Fragment match tolerance used for search: 0.02 Da
Fragments used for search: a; a-H_2O; a-NH_3; b; b-H_2O; b-NH_3; y; y-H_2O; y-NH_3
Protein references (5):
- Histone H3.1t OS=Homo sapiens GN=HIST3H3 PE=1 SV=3 - [H31T_HUMAN]
- Histone H3.1 OS=Homo sapiens GN=HIST1H3A PE=1 SV=2 - [H31_HUMAN]
- Histone H3.2 OS=Homo sapiens GN=HIST2H3A PE=1 SV=3 - [H32_HUMAN]
- Histone H3.3 OS=Homo sapiens GN=H3F3A PE=1 SV=2 - [H33_HUMAN]
- Histone H3.3C OS=Homo sapiens GN=H3F3C PE=1 SV=3 - [H3C_HUMAN]

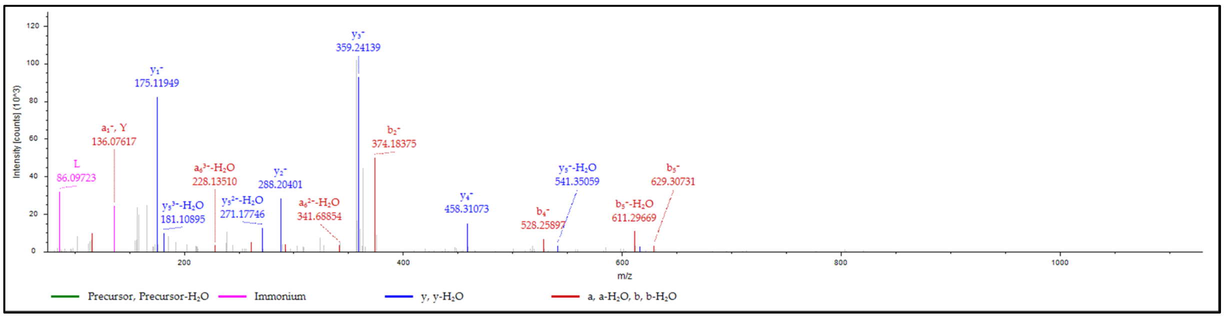

#1	Immonium	a⁺	a²⁺	a³⁺	b⁺	b²⁺	b³⁺	Seq.	y⁺	y²⁺	y³⁺	#2
1	136.07568	136.07568	68.54148	46.03008	164.07060	82.53894	55.36172	Y				9
2	129.11348	346.18737	173.59732	116.06731	374.18228	187.59478	125.39894	R-MG-H1	923.54218	462.27473	308.51891	8
3	70.06513	443.24014	222.12371	148.41823	471.23505	236.12116	157.74987	P	713.43049	357.21888	238.48168	7
4	30.03383	500.26161	250.63444	167.42539	528.25652	264.63190	176.75702	G	616.37772	308.69250	206.13076	6
5	74.06004	601.30929	301.15828	201.10795	629.30420	315.15574	210.43958	T	559.35625	280.18176	187.12360	5
6	72.08078	700.37771	350.69249	234.13075	728.37262	364.68995	243.46239	V	458.30857	229.65792	153.44104	4
7	44.04948	771.41483	386.21105	257.80979	799.40974	400.20851	267.14143	A	359.24015	180.12371	120.41823	3
8	86.09643	884.49890	442.75309	295.50448	912.49381	456.75054	304.83612	L	288.20303	144.60515	96.73919	2
9								R	175.11896	88.06312	59.04450	1

Sequence: ERGGVSLAALKK, R2-MG-H1 (54.01057 Da)
Charge: +2, Monoisotopic m/z: 641.88165 Da (+4.1 mmu/+6.39 ppm), MH+: 1282.75603 Da, RT: 12.49 min,
Identified with: Sequest HT (v1.3); XCorr:2.04, Ions matched by search engine: 0/0
Fragment match tolerance used for search: 0.02 Da
Fragments used for search: a; a-H_2O; a-NH_3; b; b-H_2O; b-NH_3; y; y-H_2O; y-NH_3
Protein references (1):
- Histone H1.1 OS=Homo sapiens GN=HIST1H1A PE=1 SV=3 - [H11_HUMAN]

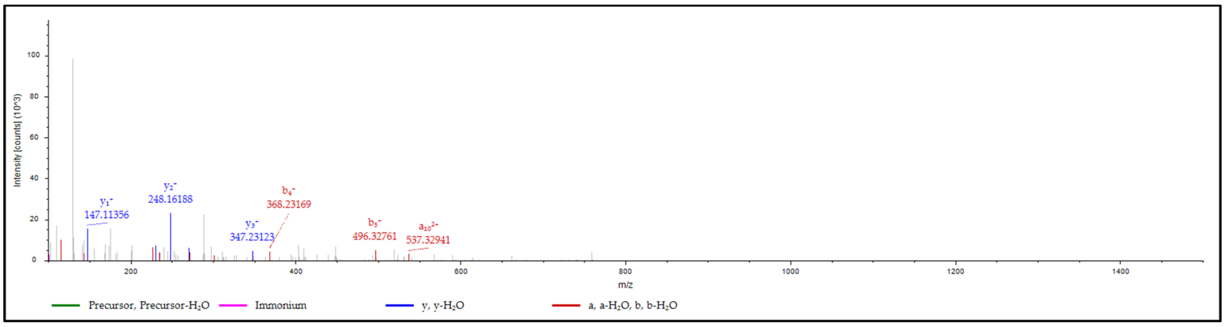

#1	Immonium	a⁺	a²⁺	a³⁺	a⁴⁺	b⁺	b²⁺	b³⁺	b⁴⁺	Seq.	y⁺	y²⁺	y³⁺	y⁴⁺	#2
1	44.04948	44.04948	22.52838	15.35468	11.76783	72.04440	36.52584	24.68632	18.76656	A					13
2	44.04948	115.08660	58.04694	39.03372	29.52711	143.08152	72.04440	48.36536	36.52584	A	1376.82608	688.91668	459.61354	344.96198	12
3	101.10733	243.18157	122.09442	81.73204	61.55085	271.17649	136.09188	91.06368	68.54958	K	1305.78896	653.39812	435.93450	327.20270	11
4	70.06513	340.23434	170.62081	114.08297	85.81404	368.22926	184.61827	123.41460	92.81277	P	1177.69399	589.35063	393.23618	295.17896	10
5	101.10733	468.32931	234.66829	156.78129	117.83779	496.32423	248.66575	166.11293	124.83651	K	1080.64122	540.82425	360.88526	270.91576	9
6	60.04439	555.36134	278.18431	185.79197	139.59579	583.35626	292.18177	195.12360	146.59452	S	952.54625	476.77676	318.18693	238.89202	8
7	30.03383	612.38281	306.69504	204.79912	153.85116	640.37773	320.69250	214.13076	160.84989	G	865.51422	433.26075	289.17626	217.13401	7
8	101.10733	794.48835	397.74781	265.50097	199.37754	822.48326	411.74527	274.83260	206.37627	K-MG-H1	808.49275	404.75001	270.16910	202.87865	6
9	70.06513	891.54112	446.27420	297.85189	223.64074	919.53603	460.27165	307.18353	230.63947	P	626.38722	313.69725	209.46726	157.35226	5
10	101.10733	1073.64665	537.32696	358.55374	269.16712	1101.64157	551.32442	367.88537	276.16585	K-MG-H1	529.33445	265.17086	177.11633	133.08907	4
11	72.08078	1172.71507	586.86117	391.57654	293.93423	1200.70999	600.85863	400.90818	300.93295	V	347.22891	174.11809	116.41449	87.56269	3
12	74.06004	1273.76275	637.38501	425.25910	319.19615	1301.75767	651.38247	434.59074	326.19487	T	248.16049	124.58388	83.39168	62.79558	2
13										K	147.11281	74.06004	49.70912	37.53366	1

Sequence: RVTIMPK, R1-MG-H1 (54.01057 Da), M5-Oxidation (15.99492 Da)
Charge: +2, Monoisotopic m/z: 457.76334 Da (+3.28 mmu/+7.16 ppm), MH+: 914.51940 Da, RT: 5.68 min,
Identified with: Sequest HT (v1.3); XCorr:2.06, Ions matched by search engine: 0/0
Fragment match tolerance used for search: 0.02 Da
Fragments used for search: a; a-H_2O; a-NH_3; b; b-H_2O; b-NH_3; y; y-H_2O; y-NH_3
Protein references (5):
- Histone H3.1t OS=Homo sapiens GN=HIST3H3 PE=1 SV=3 - [H31T_HUMAN]
- Histone H3.1 OS=Homo sapiens GN=HIST1H3A PE=1 SV=2 - [H31_HUMAN]
- Histone H3.2 OS=Homo sapiens GN=HIST2H3A PE=1 SV=3 - [H32_HUMAN]
- Histone H3.3 OS=Homo sapiens GN=H3F3A PE=1 SV=2 - [H33_HUMAN]
- Histone H3.3C OS=Homo sapiens GN=H3F3C PE=1 SV=3 - [H3C_HUMAN]

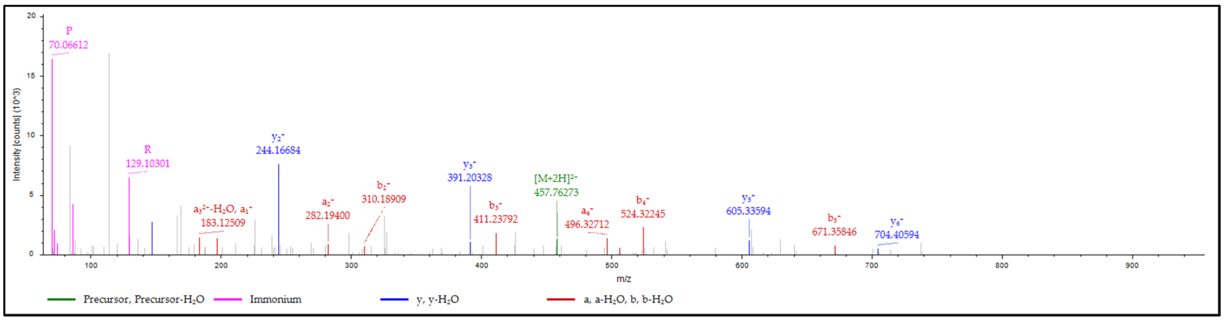

#1	Immonium	a⁺	a²⁺	b⁺	b²⁺	Seq.	y⁺	y²⁺	#2
1	129.11348	183.12405	92.06566	211.11896	106.06312	R-MG-H1			7
2	72.08078	282.19247	141.59987	310.18738	155.59733	V	704.40117	352.70422	6
3	74.06004	383.24015	192.12371	411.23506	206.12117	T	605.33275	303.17001	5
4	86.09643	496.32422	248.66575	524.31913	262.66320	I	504.28507	252.64617	4
5	104.05286	643.35963	322.18345	671.35455	336.18091	M-Oxidation	391.20100	196.10414	3
6	70.06513	740.41240	370.70984	768.40732	384.70730	P	244.16558	122.58643	2
7						K	147.11281	74.06004	1

Sequence: AGLQFPVGR, R9-MG-H1 (54.01057 Da)
Charge: +2,   Monoisotopic m/z: 499.77774 Da (+3.18 mmu/+6.37 ppm),   MH+: 998.54820 Da,   RT: 14.46 min,
Identified with: Sequest HT (v1.3); XCorr:2.28, Ions matched by search engine: 0/0
Fragment match tolerance used for search: 0.02 Da
Fragments used for search: a; a-NH_3; b; b-NH_3; y; y-NH_3

Protein references (15):
- Histone H2A type 1-A OS=Homo sapiens GN=HIST1H2AA PE=1 SV=3 - [H2A1A_HUMAN]
- Histone H2A type 1-B/E OS=Homo sapiens GN=HIST1H2AB PE=1 SV=2 - [H2A1B_HUMAN]
- Histone H2A type 1-C OS=Homo sapiens GN=HIST1H2AC PE=1 SV=3 - [H2A1C_HUMAN]
- Histone H2A type 1-D OS=Homo sapiens GN=HIST1H2AD PE=1 SV=2 - [H2A1D_HUMAN]
- Histone H2A type 1-H OS=Homo sapiens GN=HIST1H2AH PE=1 SV=3 - [H2A1H_HUMAN]
- Histone H2A type 1-J OS=Homo sapiens GN=HIST1H2AJ PE=1 SV=3 - [H2A1J_HUMAN]
- Histone H2A type 1 OS=Homo sapiens GN=HIST1H2AG PE=1 SV=2 - [H2A1_HUMAN]
- Histone H2A type 2-A OS=Homo sapiens GN=HIST2H2AA3 PE=1 SV=3 - [H2A2A_HUMAN]
- Histone H2A type 2-B OS=Homo sapiens GN=HIST2H2AB PE=1 SV=3 - [H2A2B_HUMAN]
- Histone H2A type 2-C OS=Homo sapiens GN=HIST2H2AC PE=1 SV=4 - [H2A2C_HUMAN]
- Histone H2A type 3 OS=Homo sapiens GN=HIST3H2A PE=1 SV=3 - [H2A3_HUMAN]
- Histone H2A.J OS=Homo sapiens GN=H2AFJ PE=1 SV=1 - [H2AJ_HUMAN]

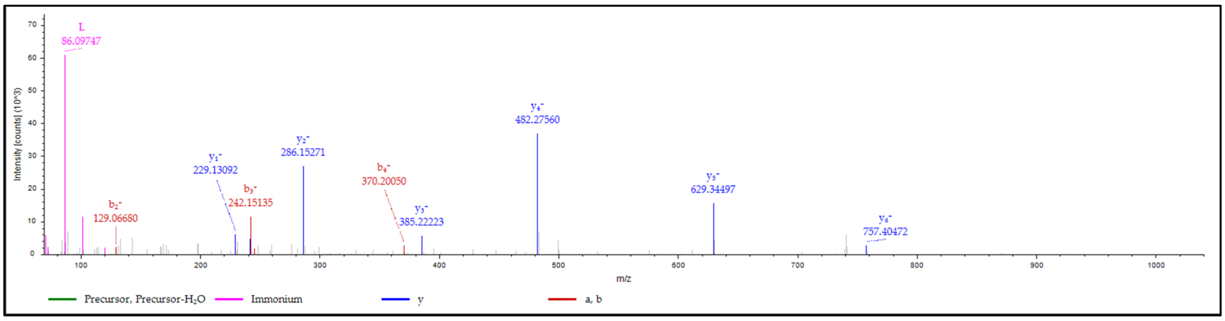

#1	Immonium	a⁺	a²⁺	b⁺	b²⁺	Seq.	y⁺	y²⁺	#2
1	44.04948	44.04948	22.52838	72.04440	36.52584	A			9
2	30.03383	101.07095	51.03911	129.06587	65.03657	G	927.50473	464.25600	8
3	86.09643	214.15502	107.58115	242.14994	121.57861	L	870.48326	435.74527	7
4	101.07094	342.21360	171.61044	370.20852	185.60790	Q	757.39919	379.20323	6
5	120.08078	489.28202	245.14465	517.27694	259.14211	F	629.34061	315.17394	5
6	70.06513	586.33479	293.67103	614.32971	307.66849	P	482.27219	241.63973	4
7	72.08078	685.40321	343.20524	713.39813	357.20270	V	385.21942	193.11335	3
8	30.03383	742.42468	371.71598	770.41960	385.71344	G	286.15100	143.57914	2
9						R-MG-H1	229.12953	115.06840	1

Sequence: DNIQGITKPAIRR, R12-MG-H1 (54.01057 Da)
Charge: +3, Monoisotopic m/z: 512.63013 Da (+3.51 mmu/+6.84 ppm), MH+: 1535.87583 Da, RT: 9.09 min,
Identified with: Sequest HT (v1.3); XCorr:3.79, Ions matched by search engine: 0/0
Fragment match tolerance used for search: 0.02 Da
Fragments used for search: a; a-H_2O; a-NH_3; b; b-H_2O; b-NH_3; y; y-H_2O; y-NH_3
Protein references (1):
- Histone H4 OS=Homo sapiens GN=HIST1H4A PE=1 SV=2 - [H4_HUMAN]

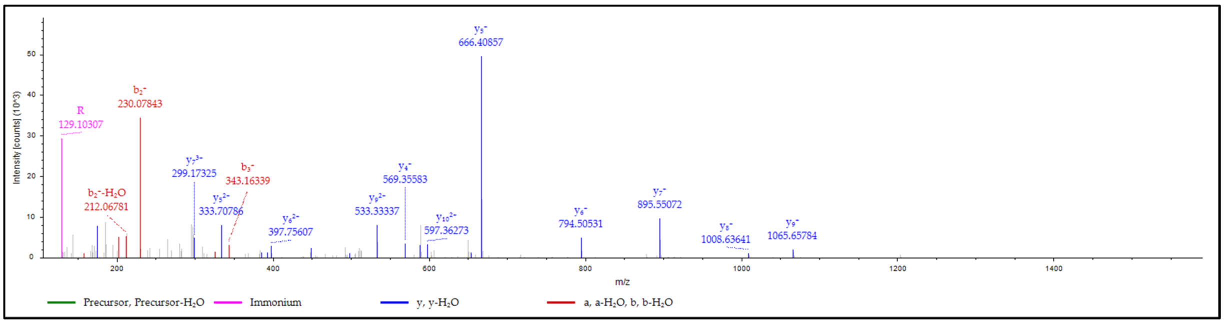

#1	Immonium	a⁺	a²⁺	a³⁺	b⁺	b²⁺	b³⁺	Seq.	y⁺	y²⁺	y³⁺	#2
1	88.03931	88.03931	44.52329	30.01796	116.03423	58.52075	39.34959	D				13
2	87.05529	202.08224	101.54476	68.03227	230.07716	115.54222	77.36390	N	1420.83838	710.92283	474.28431	12
3	86.09643	315.16631	158.08679	105.72696	343.16123	172.08425	115.05859	I	1306.79545	653.90136	436.27000	11
4	101.07094	443.22489	222.11608	148.41315	471.21981	236.11354	157.74479	Q	1193.71138	597.35933	398.57531	10
5	30.03383	500.24636	250.62682	167.42031	528.24128	264.62428	176.75194	G	1065.65280	533.33004	355.88912	9
6	86.09643	613.33043	307.16885	205.11500	641.32535	321.16631	214.44663	I	1008.63133	504.81930	336.88196	8
7	74.06004	714.37811	357.69269	238.79756	742.37303	371.69015	248.12919	T	895.54726	448.27727	299.18727	7
8	101.10733	842.47308	421.74018	281.49588	870.46800	435.73764	290.82752	K	794.49958	397.75343	265.50471	6
9	70.06513	939.52585	470.26656	313.84680	967.52077	484.26402	323.17844	P	666.40461	333.70594	222.80639	5
10	44.04948	1010.56297	505.78512	337.52584	1038.55789	519.78258	346.85748	A	569.35184	285.17956	190.45546	4
11	86.09643	1123.64704	562.32716	375.22053	1151.64196	576.32462	384.55217	I	498.31472	249.66100	166.77642	3
12	129.11348	1333.75873	667.38300	445.25776	1361.75364	681.38046	454.58940	R-MG-H1	385.23065	193.11896	129.08173	2
13								R	175.11896	88.06312	59.04450	1

Sequence: RVTIMPK, R1-MG-H1 (54.01057 Da), M5-Oxidation (15.99492 Da)
Charge: +2, Monoisotopic m/z: 457.76288 Da (+2.82 mmu/+6.16 ppm), MH+: 914.51848 Da, RT: 5.78 min,
Identified with: Sequest HT (v1.3); XCorr:1.98, Ions matched by search engine: 0/0
Fragment match tolerance used for search: 0.02 Da
Fragments used for search: a; a-H_2O; a-NH_3; b; b-H_2O; b-NH_3; y; y-H_2O; y-NH_3

Protein references (5):
- Histone H3.1t OS=Homo sapiens GN=HIST3H3 PE=1 SV=3 - [H31T_HUMAN]
- Histone H3.1 OS=Homo sapiens GN=HIST1H3A PE=1 SV=2 - [H31_HUMAN]
- Histone H3.2 OS=Homo sapiens GN=HIST2H3A PE=1 SV=3 - [H32_HUMAN]
- Histone H3.3 OS=Homo sapiens GN=H3F3A PE=1 SV=2 - [H33_HUMAN]
- Histone H3.3C OS=Homo sapiens GN=H3F3C PE=1 SV=3 - [H3C_HUMAN]

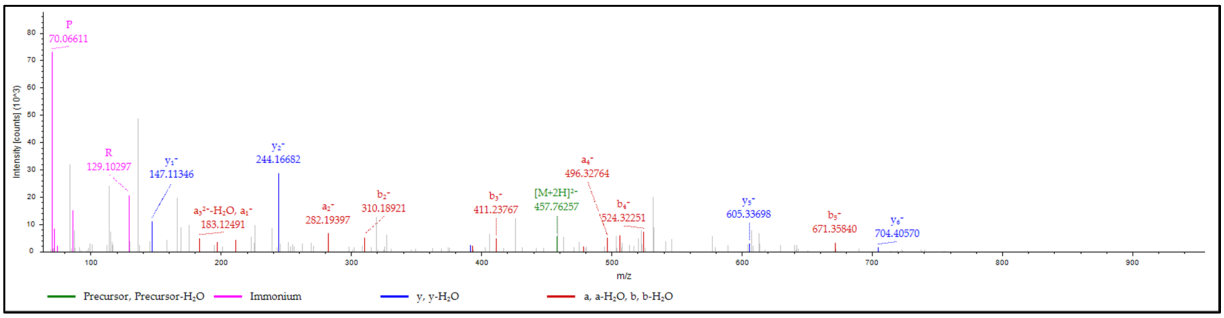

#1	Immonium	a⁺	a²⁺	b⁺	b²⁺	Seq.	y⁺	y²⁺	#2
1	129.11348	183.12405	92.06566	211.11896	106.06312	R-MG-H1			7
2	72.08078	282.19247	141.59987	310.18738	155.59733	V	704.40117	352.70422	6
3	74.06004	383.24015	192.12371	411.23506	206.12117	T	605.33275	303.17001	5
4	86.09643	496.32422	248.66575	524.31913	262.66320	I	504.28507	252.64617	4
5	104.05286	643.35963	322.18345	671.35455	336.18091	M-Oxidation	391.20100	196.10414	3
6	70.06513	740.41240	370.70984	768.40732	384.70730	P	244.16558	122.58643	2
7						K	147.11281	74.06004	1

Sequence: AGLQFPVGR, R9-MG-H1 (54.01057 Da)
Charge: +2, Monoisotopic m/z: 499.77725 Da (+2.69 mmu/+5.39 ppm), MH+: 998.54723 Da, RT: 14.53 min,
Identified with: Sequest HT (v1.3); XCorr:2.19, Ions matched by search engine: 0/0
Fragment match tolerance used for search: 0.02 Da
Fragments used for search: a; a-NH_3; b; b-NH_3; y; y-NH_3

Protein references (15):
- Histone H2A type 1-A OS=Homo sapiens GN=HIST1H2AA PE=1 SV=3 - [H2A1A_HUMAN]
- Histone H2A type 1-B/E OS=Homo sapiens GN=HIST1H2AB PE=1 SV=2 - [H2A1B_HUMAN]
- Histone H2A type 1-C OS=Homo sapiens GN=HIST1H2AC PE=1 SV=3 - [H2A1C_HUMAN]
- Histone H2A type 1-D OS=Homo sapiens GN=HIST1H2AD PE=1 SV=2 - [H2A1D_HUMAN]
- Histone H2A type 1-H OS=Homo sapiens GN=HIST1H2AH PE=1 SV=3 - [H2A1H_HUMAN]
- Histone H2A type 1-J OS=Homo sapiens GN=HIST1H2AJ PE=1 SV=3 - [H2A1J_HUMAN]
- Histone H2A type 1 OS=Homo sapiens GN=HIST1H2AG PE=1 SV=2 - [H2A1_HUMAN]
- Histone H2A type 2-A OS=Homo sapiens GN=HIST2H2AA3 PE=1 SV=3 - [H2A2A_HUMAN]
- Histone H2A type 2-B OS=Homo sapiens GN=HIST2H2AB PE=1 SV=3 - [H2A2B_HUMAN]
- Histone H2A type 2-C OS=Homo sapiens GN=HIST2H2AC PE=1 SV=4 - [H2A2C_HUMAN]
- Histone H2A type 3 OS=Homo sapiens GN=HIST3H2A PE=1 SV=3 - [H2A3_HUMAN]
- Histone H2A.J OS=Homo sapiens GN=H2AFJ PE=1 SV=1 - [H2AJ_HUMAN]
- Histone H2A.V OS=Homo sapiens GN=H2AFV PE=1 SV=3 - [H2AV_HUMAN]
- Histone H2AX OS=Homo sapiens GN=H2AFX PE=1 SV=2 - [H2AX_HUMAN]
- Histone H2A.Z OS=Homo sapiens GN=H2AFZ PE=1 SV=2 - [H2AZ_HUMAN]

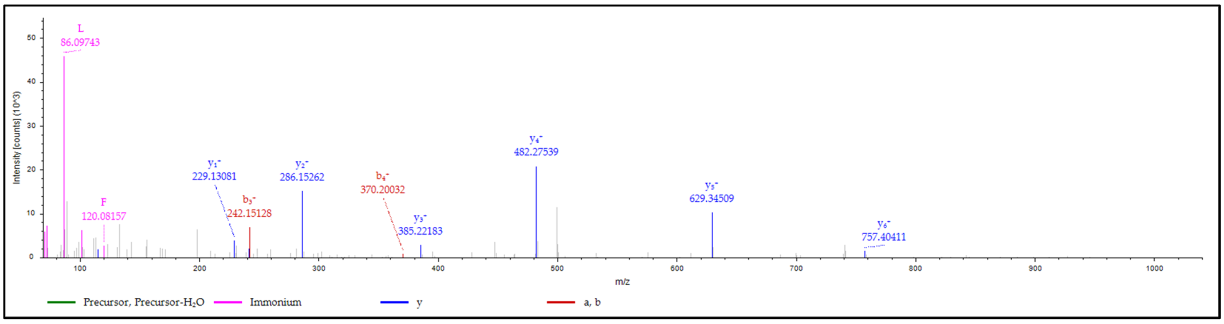

#1	Immonium	a⁺	a²⁺	b⁺	b²⁺	Seq.	y⁺	y²⁺	#2
1	44.04948	44.04948	22.52838	72.04440	36.52584	A			9
2	30.03383	101.07095	51.03911	129.06587	65.03657	G	927.50473	464.25600	8
3	86.09643	214.15502	107.58115	242.14994	121.57861	L	870.48326	435.74527	7
4	101.07094	342.21360	171.61044	370.20852	185.60790	Q	757.39919	379.20323	6
5	120.08078	489.28202	245.14465	517.27694	259.14211	F	629.34061	315.17394	5
6	70.06513	586.33479	293.67103	614.32971	307.66849	P	482.27219	241.63973	4
7	72.08078	685.40321	343.20524	713.39813	357.20270	V	385.21942	193.11335	3
8	30.03383	742.42468	371.71598	770.41960	385.71344	G	286.15100	143.57914	2
9						R-MG-H1	229.12953	115.06840	1

Sequence: RVTIMPK, R1-MG-H1 (54.01057 Da), M5-Oxidation (15.99492 Da)
Charge: +2, Monoisotopic m/z: 457.76324 Da (+3.18 mmu/+6.96 ppm), MH+: 914.51921 Da, RT: 5.81 min,
Identified with: Sequest HT (v1.3); XCorr:2.01, Ions matched by search engine: 0/0
Fragment match tolerance used for search: 0.02 Da
Fragments used for search: a; a-H_2O; a-NH_3; b; b-H_2O; b-NH_3; y; y-H_2O; y-NH_3
Protein references (5):
- Histone H3.1t OS=Homo sapiens GN=HIST3H3 PE=1 SV=3 - [H31T_HUMAN]
- Histone H3.1 OS=Homo sapiens GN=HIST1H3A PE=1 SV=2 - [H31_HUMAN]
- Histone H3.2 OS=Homo sapiens GN=HIST2H3A PE=1 SV=3 - [H32_HUMAN]
- Histone H3.3 OS=Homo sapiens GN=H3F3A PE=1 SV=2 - [H33_HUMAN]
- Histone H3.3C OS=Homo sapiens GN=H3F3C PE=1 SV=3 - [H3C_HUMAN]

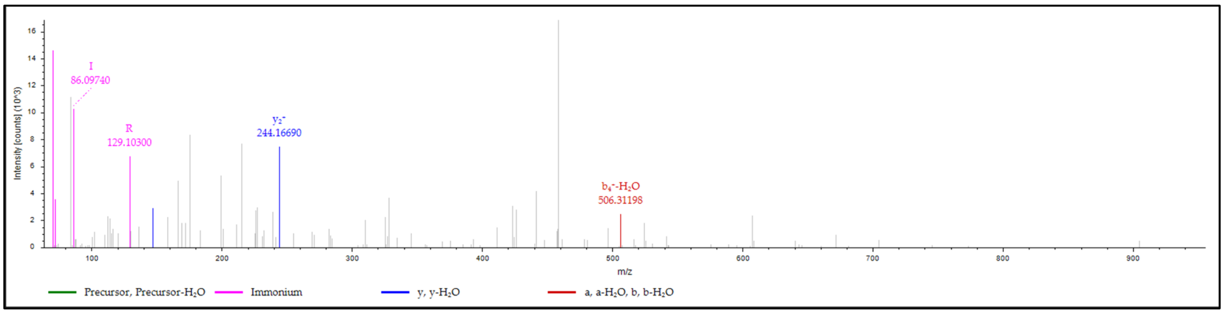

#1	Immonium	a⁺	a²⁺	b⁺	b²⁺	Seq.	y⁺	y²⁺	#2
1	129.11348	183.12405	92.06566	211.11896	106.06312	R-MG-H1			7
2	72.08078	282.19247	141.59987	310.18738	155.59733	V	704.40117	352.70422	6
3	74.06004	383.24015	192.12371	411.23506	206.12117	T	605.33275	303.17001	5
4	86.09643	496.32422	248.66575	524.31913	262.66320	I	504.28507	252.64617	4
5	104.05286	643.35963	322.18345	671.35455	336.18091	M-Oxidation	391.20100	196.10414	3
6	70.06513	740.41240	370.70984	768.40732	384.70730	P	244.16558	122.58643	2
7						K	147.11281	74.06004	1

Sequence: KAGAAKPR, K1-MG-H1 (54.01057 Da),
Charge: +2, Monoisotopic m/z: 426.75247 Da (+2.16 mmu/+1.23 ppm), MH+: 852.49767 Da, RT: 11.07 min,
Identified with: Sequest HT (v1.3); XCorr:2.01, Ions matched by search engine: 0/0
Fragment match tolerance used for search: 0.02 Da
Fragments used for search: a; a-H_2O; a-NH_3; b; b-H_2O; b-NH_3; y; y-H_2O; y-NH_3

Protein references(1)

- Histone H1.3t OS=Homo sapiens GN= HIST1H1D PE=1 SV=3 - [H13_HUMAN]

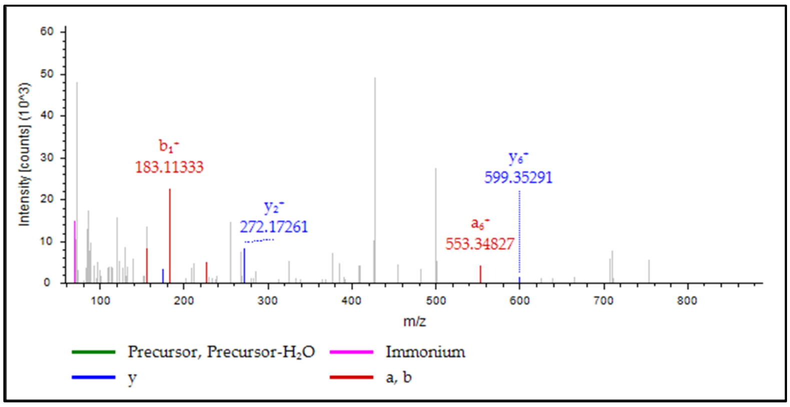

#1	Immonium	a⁺	a²⁺	b⁺	b²⁺	Seq.	y⁺	y²⁺	#2
1	101.10733	155.11790	78.06259	183.11281	92.06004	K-MG-H1			8
2	44.04948	226.15502	113.58115	254.14993	127.57860	A	670.39953	335.70340	7
3	30.03383	283.17649	142.09188	311.17140	156.08934	G	599.36241	300.18484	6
4	44.04948	354.21361	177.61044	382.20852	191.60790	A	542.34094	271.67411	5
5	44.04948	425.25073	213.12900	453.24564	227.12646	A	471.30382	236.15555	4
6	101.10733	553.34570	277.17649	581.34061	291.17394	K	400.26670	200.63699	3
7	70.06513	650.39847	325.70287	678.39338	339.70033	P	272.17173	136.58950	2
8						R	175.11896	88.06312	1
