# Supplementary material for: DJ-1 Proteoforms in Breast Cancer Cells: The Escape of Metabolic Epigenetic Misregulation
Source: Cells. 2020 Aug 26;9(9):1968. doi: 10.3390/cells9091968 (PMC7563694; doi:10.3390/cells9091968)
Supplement: Supplementary file 1 [file cells-09-01968-s001.zip › SI_03/Supplemtary file 4.pdf]

- Home
- News

FEATURES
- Scan Protein for Motifs
- Search a Sequence Database for Motifs
- Find Sequence Match
- Scan Orthologs
- Predict Localization
- Calculate Molecular Weight and pI
- Calculate Amino Acid Composition
- Databases and Motifs

TUTORIALS
- Tutorial Overview
- Protein Scan
- Database Search
- Sequence Match

You are here: Scansite 4.0 › Features › Scan Orthologs › Result

Scan for evolutionary conserved phosphorylation sites - Result

Scan properties

ORTHOLOGY DATABASE	SwissProt Orthology
PROTEIN DATABASE	UniProtKB/Swiss-Prot
PROTEIN / GENE IDENTIFIER	PARK7_HUMAN
SITE PREDICTION STRINGENCY	Minimum
SEQUENCE ALIGNMENT RADIUS	40
MOTIF GROUP	Basophilic serine/threonine kinase group
SITE POSITION IN QUERY PROTEIN	124

Scan result properties

NUMBER OF ORTHOLOGOUS PROTEINS	8
NUMBER OF ORTHOLOGOUS PROTEINS WITH CONSERVED PHOSPHORYLATION SITE	8
EVOLUTIONARY CONSERVATION OF PHOSPHORYLATION SITE	100%

Orthologous proteins

Warning: Popup blockers may disable the links in the result table

Protein ID	Protein Annotations	Molecular Weight	Isoelectric Point	Motifs at expected sites
PARK7_RAT	RecName: Full=Protein/nucleic acid de (click to expand)	19976.857	6.323	Akt_Kin
PARK7_MOUSE	RecName: Full=Protein/nucleic acid de (click to expand)	20023.990	6.320	Akt_Kin
PARK7_MESAU	RecName: Full=Protein/nucleic acid de (click to expand)	19917.729	6.326	Akt_Kin
PARK7_HUMAN	RecName: Full=Protein/nucleic acid de (click to expand)	19893.738	6.329	Akt_Kin
PARK7_DANRE	RecName: Full=Protein/nucleic acid de (click to expand)	19766.584	5.838	Casn_Kin1, Hrr25, Yck1_Yck2_Yck3
PARK7_CHLAE	RecName: Full=Protein/nucleic acid de (click to expand)	19907.764	6.329	Akt_Kin
PARK7_CHICK	RecName: Full=Protein/nucleic acid de (click to expand)	19945.846	6.325	1433_m1, Akt_Kin, phosphosite-PKCA
PARK7_BOVIN	RecName: Full=Protein/nucleic acid de (click to expand)	20037.995	6.841	Akt_Kin
Protein ID	Protein Annotations	Molecular Weight	Isoelectric Point	Motifs at expected sites

Sequence alignment

```
PARK7_BOVIN|                                      Bos taurus (Bovine)|SAAVKEILKEQEKRKGLIAAICAGPTALLAHEIGFGSKVTTHPLAKDKMMNGSHYSYSENRVEKDGLILTSRGPGTSFEF
PARK7_CHICK|                                  Gallus gallus (Chicken)|SAAVKDILKDQESRKGLIAAICAGPTALLAHGIGFGSKVITHPLAKDKMMNGAHYCYSESRVEKDGNILTSRGPGTSFEF
PARK7_CHLAE|Chlorocebus aethiops (Green monkey) (Cercopithecus aethiops)|SAAVKEILKEQENRKGLIAAICAGPTALLAHEIGFGSKVTTHPLAKDKMMNGGHYTYSENRVEKDGLILTSRGPGTSFEF
PARK7_DANRE|                Danio rerio (Zebrafish) (Brachydanio rerio)|SPAVKEVLKDQEGRKGLIAAICAGPTALLAHGIAYGSTVTTHPGAKDKMMAGDHYKYSEARVQKDGNVITSRGPGTSFEF
PARK7_HUMAN|                                      Homo sapiens (Human)|SAAVKEILKEQENRKGLIAAICAGPTALLAHEIGFGSKVTTHPLAKDKMMNGGHYTYSENRVEKDGLILTSRGPGTSFEF
PARK7_MESAU|                    Mesocricetus auratus (Golden hamster)|SPVVKEILKEQESRKGLIAAICAGPTALLAHEIGFGSKVTTHPGAKDKMMNGSHYSYSESRVEKDGLILTSRGPGTSFEF
PARK7_MOUSE|                                      Mus musculus (Mouse)|SPMVKEILKEQESRKGLIAAICAGPTALLAHEVGFGCKVTTHPLAKDKMMNGSHYSYSESRVEKDGLILTSRGPGTSFEF
  PARK7_RAT|                                  Rattus norvegicus (Rat)|SALVKEILKEQENRKGLIAAICAGPTALLAHEVGFGCKVTSHPLAKDKMMNGSHYSYSESRVEKDGLILTSRGPGTSFEF
```


© 2020 - Scansite 4.00.023 - Michael B. Yaffe Laboratory - Koch Institute, MIT
