# Supplementary material for: DJ-1 Proteoforms in Breast Cancer Cells: The Escape of Metabolic Epigenetic Misregulation
Source: Cells. 2020 Aug 26;9(9):1968. doi: 10.3390/cells9091968 (PMC7563694; doi:10.3390/cells9091968)
Supplement: Supplementary file 1 [file cells-09-01968-s001.zip › SI_03/Supplemtary file 6.pdf]

A

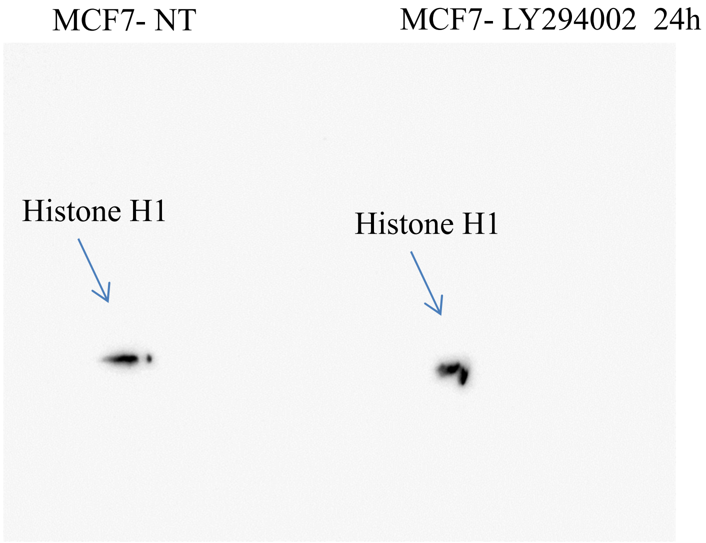

B

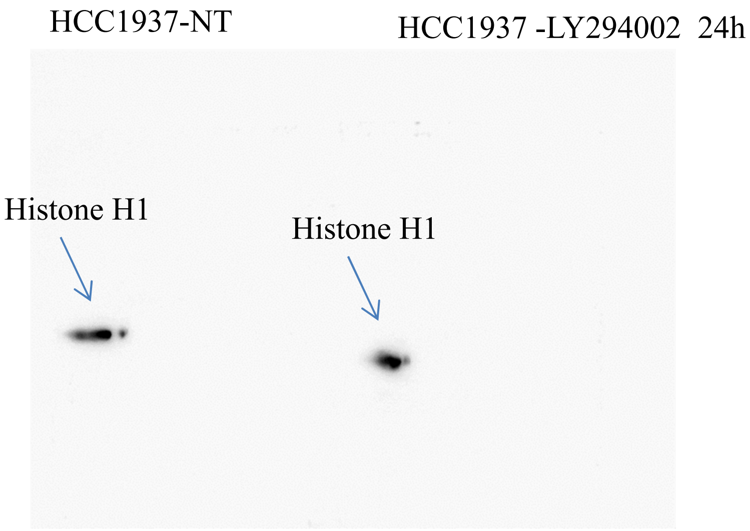

C

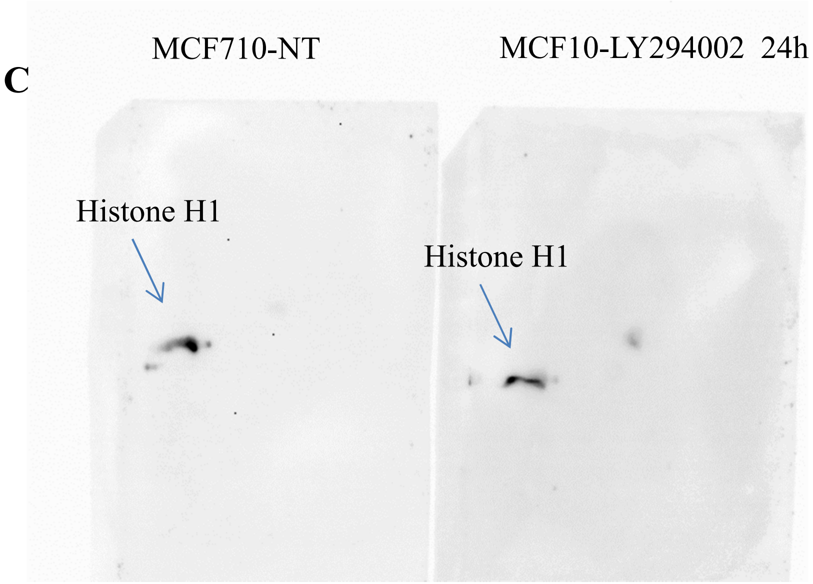

2D TAU Western Blot Normalization

2D TAU western blots showing the levels of Histone H1 in MCF7 (A), HCC1937(B) and MCF10 (C)cells.

Histone H1 was used as loading control.

Equal amounts of histone extracts were resolved by 2D TAU/SDS gel. To minimize gel to gel variation and ensure a reliable comparison among analyzed samples second dimension for each sample was run on Mini-PROTEAN® TGX™ Precast Gels, IPG Well. Resulting gel were transferred to nitrocellulose membranes with a Trans-blot turbo system (Biorad) using Trans-Blot® Turbo™ Mini Nitrocellulose Transfer Packs.

Membrane were stained with red ponceau solution (Panel D)

Membranes were hybridized with the antibody against H1(ab 71594).

To ensure equal protein loading, Blot images were acquired using Alliance 2.7 (UVITEC, Eppendorf, Milan, Italy). Signals for treated and untreated cells were acquired concomitantly at 4 seconds.

D RED PONCEAU

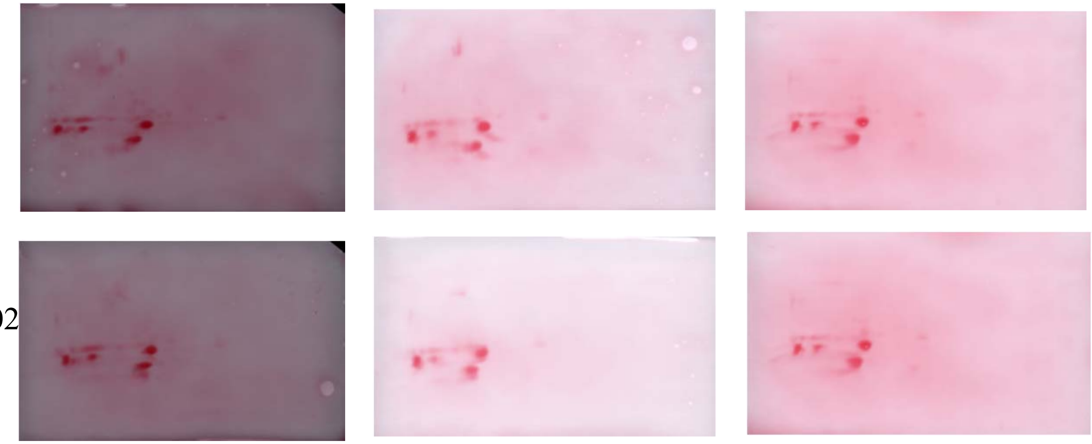

E 3d VIEW

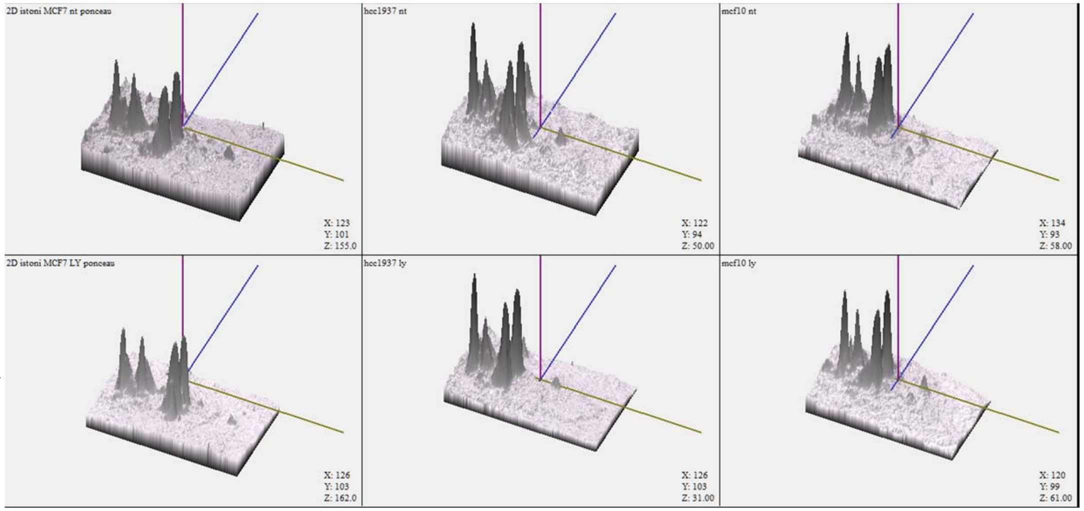

D) In the panel are shown uncropped Red ponceau Staining of membranes for HCC1937, MCF7 and MCF10 cell lines.
For 2D TAU Western blot analysis, equal amounts of histone extracts were resolved by 2D TAU/SDS gel and blotted as previously reported. To further ensure equal protein loading, membranes were incubated with red ponceau solution (P7170 Sigma-Aldrich).
E) Images were acquired using Image scanner II (GE Healthcare).3D visualization tool testify the loading of equal amount for each Histone extract in all analyzed cell lines. Staining and Image master analysis were done either in LY294002 treated cells than in untreated .
